# Supplementary material for: Synthesis of Novel α-Trifluoroanisole Derivatives Containing Phenylpyridine Moieties with Herbicidal Activity
Source: Int J Mol Sci. 2022 Sep 21;23(19):11083. doi: 10.3390/ijms231911083 (PMC9570041; doi:10.3390/ijms231911083)
Supplement: Supplementary file 1 [file ijms-23-11083-s001.zip › ijms-1893332-supplementary.pdf]

Synthesis of novel α-trifluoroanisole derivatives containing phenylpyridine moieties with herbicidal activity

Zengfei Cai, Yangyang Cao, Xiaohua Du*

Catalytic Hydrogenation Research Center, Zhejiang Key Laboratory of Green Pesticides and Cleaner Production Technology, Zhejiang Green Pesticide Collaborative Innovation Center, Zhejiang University of Technology, Hangzhou 310014, P. R. China

*Corresponding author.
E-mail addresses: duxiaohua@zjut.edu.cn

Supporting Information

Contents:

1. Data of **7a** in Single-crystal X-ray Diffraction 2

2. ^{1}H NMR, ^{13}C NMR, and HRMS spectra of target compounds **7a-7r** 17

1. Data of 7a in Single-crystal X-ray Diffraction

Table S1. Crystal data and structure refinement parameters of compound 7a

Parameter	Value	
Empirical formula	$C_{20}H_{12}ClF_6NO_2$	
Formula weight	447.09	
Temperature	296 K	
Wavelength	1.34139 Å	
Crystal system	Monoclinic	
Space group	P_121/c_1	
	a = 9.8946(7) Å	α = 90°
Unit cell dimensions	b = 40.191(3) Å	β = 102.295(3)°
	c = 9.9891(8) Å	γ = 90°
Volume	3881.3(5) Å^3	
Z	8	
Density (calculated)	1.530 Mg/m^3	
Absorption coefficient	1.575 mm^{-1}	
F(000)	1805	
Crystal size	0.05 × 0.02 × 0.02 mm^3	
Theta range for data collection	3.827° to 54.900°	
Index ranges	$-12 \le h \le 8$, $-49 \le k \le 49$, $-12 \le l \le 12$	
Reflections collected	33681	
Independent reflections	7256 [R(int) = 0.0542]	
Completeness to theta = 53.594°	98.70%	
Absorption correction	Semi-empirical from equivalents	

Max. and min. transmission	0.7508 and 0.6181
Refinement method	Full-matrix least-squares on F^2
Data / restraints / parameters	7256 / 0 / 627
Goodness-of-fit on F^2	1.02
Final R indices [$I > 2\text{sigma}(I)$]	$R_1 = 0.0894$, $\omega R_2 = 0.2127$
R indices (all data)	$R_1 = 0.1627$, $\omega R_2 = 0.2600$
Extinction coefficient	None
Largest diff. peak and hole	0.481 and -0.318 e.Å^{-3}

Crystal structure determination of 7a

Crystal Data. $C_{20}H_{12}ClF_6NO_2$ (M =447.09 g/mol): monoclinic, space group P 1 2_1/c 1 , a = 9.8946(7) Å, b = 40.191(3) Å, c = 9.9891(8) Å, β = 102.295(3)°, V = 3881.3(5) Å^3, Z = 8, T = 298 K, μ(Cu Kα) = 1.575 mm^{-1}, $Dcalc$ = 1.530 g/cm^3, 33681 reflections measured (3.827° ≤ 2θ ≤54.900°), 7256 unique (R_{int} = 0.0542) which were used in all calculations. The final R_1 was 0.0894 (I > 2σ(I)) and wR_2 was 0.2127 (all data).

Table S2. Bond angles of compound 7a

Atoms	Angle (°)	Atoms	Angle (°)
C(10)-O(1)-C(13)	117.4(4)	C(20)-O(2)-C(17)	122.0(6)
C(1)-N(1)-C(5)	119.3(4)	N(1)-C(1)-H(1)	117.7
N(1)-C(1)-C(2)	124.6(5)	C(2)-C(1)-H(1)	117.7
C(1)-C(2)-C(3)	117.6(5)	C(1)-C(2)-C(6)	120.1(5)
C(3)-C(2)-C(6)	122.4(5)	C(2)-C(3)-H(3)	120.5
C(2)-C(3)-C(4)	119.1(5)	C(4)-C(3)-H(3)	120.5
C(3)-C(4)-Cl(1)	117.1(4)	C(3)-C(4)-C(5)	121.2(4)

C(5)-C(4)-Cl(1)	121.7(4)	N(1)-C(5)-C(4)	118.3(4)
N(1)-C(5)-C(7)	115.2(4)	C(4)-C(5)-C(7)	126.5(4)
F(1)-C(6)-F(2)	102.6(7)	F(1)-C(6)-F(3)	102.4(9)
F(1)-C(6)-C(2)	117.7(6)	F(2)-C(6)-C(2)	114.4(8)
F(3)-C(6)-F(2)	101.3(7)	F(3)-C(6)-C(2)	116.1(6)
C(8)-C(7)-C(5)	119.8(4)	C(12)-C(7)-C(5)	123.1(4)
C(12)-C(7)-C(8)	117.0(4)	C(7)-C(8)-H(8)	119
C(9)-C(8)-C(7)	121.9(5)	C(9)-C(8)-H(8)	119
C(8)-C(9)-H(9)	119.8	C(8)-C(9)-C(10)	120.4(4)
C(10)-C(9)-H(9)	119.8	O(1)-C(10)-C(9)	116.3(4)
O(1)-C(10)-C(11)	124.7(4)	C(11)-C(10)-C(9)	118.9(4)
C(10)-C(11)-H(11)	120.2	C(10)-C(11)-C(12)	119.5(5)
C(12)-C(11)-H(11)	120.2	C(7)-C(12)-C(11)	122.2(4)
C(7)-C(12)-H(12)	118.9	C(11)-C(12)-H(12)	118.9
O(1)-C(13)-H(13A)	109.9	O(1)-C(13)-H(13B)	109.9
O(1)-C(13)-C(14)	108.8(4)	H(13A)-C(13)-H(13B)	108.3
C(14)-C(13)-H(13A)	109.9	C(14)-C(13)-H(13B)	109.9
C(15)-C(14)-C(13)	121.0(5)	C(15)-C(14)-C(19)	118.2(5)
C(19)-C(14)-C(13)	120.8(5)	C(14)-C(15)-H(15)	119
C(14)-C(15)-C(16)	122.1(5)	C(16)-C(15)-H(15)	119
C(15)-C(16)-H(16)	120.6	C(17)-C(16)-C(15)	118.8(5)
C(17)-C(16)-H(16)	120.6	C(16)-C(17)-O(2)	118.5(5)
C(16)-C(17)-C(18)	121.1(5)	C(18)-C(17)-O(2)	120.1(5)
C(17)-C(18)-H(18)	120.5	C(19)-C(18)-C(17)	119.0(5)

C(19)-C(18)-H(18)	120.5	C(14)-C(19)-H(19)	119.5
C(18)-C(19)-C(14)	120.9(5)	C(18)-C(19)-H(19)	119.5
F(4)-C(20)-F(6)	102.4(8)	F(5)-C(20)-F(4)	103.1(11)
F(5)-C(20)-F(6)	110.9(8)	F(5)-C(20)-O(2)	115.5(8)
O(2)-C(20)-F(4)	108.1(8)	O(2)-C(20)-F(6)	115.1(8)
C(21)-N(2)-C(25)	119.2(4)	N(2)-C(21)-H(21)	118
N(2)-C(21)-C(22)	123.9(5)	C(22)-C(21)-H(21)	118
C(21)-C(22)-C(23)	117.7(4)	C(21)-C(22)-C(26)	119.7(5)
C(23)-C(22)-C(26)	122.7(5)	C(22)-C(23)-H(23)	120.4
C(22)-C(23)-C(24)	119.3(4)	C(24)-C(23)-H(23)	120.4
C(23)-C(24)-Cl(2)	117.4(4)	C(23)-C(24)-C(25)	120.4(4)
C(25)-C(24)-Cl(2)	122.0(4)	N(2)-C(25)-C(24)	119.5(4)
N(2)-C(25)-C(27)	114.6(4)	C(24)-C(25)-C(27)	125.9(4)
F(7)-C(26)-C(22)	110.2(9)	F(9)-C(26)-C(22)	115.1(9)
F(9)-C(26)-F(7)	95.4(15)	F(9)-C(26)-F(11)	113.2(18)
F(11)-C(26)-C(22)	115.3(9)	F(11)-C(26)-F(7)	105.3(14)
F(8)-C(26)-C(22)	109.4(14)	F(10)-C(26)-C(22)	113.8(8)
F(10)-C(26)-F(8)	102(2)	F(12)-C(26)-C(22)	112.8(10)
F(12)-C(26)-F(8)	112.6(19)	F(12)-C(26)-F(10)	105.5(13)
C(28)-C(27)-C(25)	119.1(4)	C(28)-C(27)-C(32)	117.3(4)
C(32)-C(27)-C(25)	123.6(4)	C(27)-C(28)-H(28)	118.9
C(29)-C(28)-C(27)	122.3(4)	C(29)-C(28)-H(28)	118.9
C(28)-C(29)-H(29)	119.9	C(28)-C(29)-C(30)	120.2(4)
C(30)-C(29)-H(29)	119.9	C(29)-C(30)-C(31)	118.7(4)

C(29)-C(30)-O(5)	136.7(6)	C(31)-C(30)-O(5)	104.6(6)
O(3)-C(30)-C(29)	109.3(5)	O(3)-C(30)-C(31)	132.0(6)
C(30)-C(31)-H(31)	120(3)	C(32)-C(31)-C(30)	120.8(5)
C(32)-C(31)-H(31)	119(3)	C(27)-C(32)-H(32)	119.6
F(13)-C(40)-F(14)	113.9(10)	F(13)-C(40)-F(15)	104.2(10)
F(13)-C(40)-O(6)	113.2(9)	F(14)-C(40)-F(15)	106.5(10)
F(14)-C(40)-O(6)	107.4(9)	F(15)-C(40)-O(6)	111.6(9)
F(17)-C(40)-F(16)	106.9(11)	F(17)-C(40)-O(4)	118.8(11)
F(18)-C(40)-F(16)	109.0(16)	F(18)-C(40)-F(17)	102.9(18)
F(18)-C(40)-O(4)	115(2)	O(4)-C(40)-F(16)	104.2(11)
C(30)-O(3)-C(33)	112.8(7)	C(40)-O(6)-C(37)	112.8(7)
C(34)-C(33)-H(33A)	112.1	C(34)-C(33)-H(33B)	112.1
O(3)-C(33)-C(34)	98.5(7)	O(3)-C(33)-H(33A)	112.1
O(3)-C(33)-H(33B)	112.1	H(33A)-C(33)-H(33B)	109.7
C(40)-O(4)-C(37)	105.2(9)	C(41)-O(5)-C(30)	109.3(11)
C(34)-C(41)-H(41A)	113.6	C(34)-C(41)-H(41B)	113.6
O(5)-C(41)-C(34)	90.5(9)	O(5)-C(41)-H(41A)	113.6
O(5)-C(41)-H(41B)	113.6	H(41A)-C(41)-H(41B)	110.8

Table S3. Atomic coordinates (× 10^4) and equivalent isotropic displacement parameters (Å^2 × 10^3) of the nonhydrogen atoms

Atom	x	y	z	U_{eq}
Cl (1)	4881(2)	3250(1)	2604(2)	101(1)
F (1)	211(7)	2662(1)	-1931(8)	237(4)
F (2)	2069(6)	2483(2)	-1607(9)	266(5)

F (3)	973(10)	2431(1)	-252(6)	253(4)
F (4)	7501(9)	6526(2)	3274(11)	287(5)
F (5)	7665(10)	6441(2)	5193(9)	318(7)
F (6)	6603(6)	6841(1)	4300(6)	191(2)
O (1)	4324(3)	4877(1)	2333(4)	74(1)
O (2)	5787(5)	6357(1)	3866(5)	118(2)
N (1)	1724(5)	3545(1)	-323(4)	86(1)
C (1)	1259(6)	3251(1)	-824(6)	91(2)
C (2)	1864(6)	2953(1)	-390(5)	76(1)
C (3)	3004(5)	2958(1)	666(5)	74(1)
C (4)	3496(5)	3259(1)	1217(5)	66(1)
C (5)	2862(5)	3560(1)	705(4)	62(1)
C (6)	1277(10)	2643(2)	-1031(8)	114(2)
C(7)	3304(5)	3898(1)	1187(4)	61(1)
C (8)	2320(5)	4144(1)	1195(5)	72(1)
C (9)	2680(5)	4463(1)	1571(5)	75(1)
C (10)	4061(5)	4552(1)	1967(4)	60(1)
C (11)	5061(5)	4314(1)	1959(5)	71(1)
C (12)	4668(5)	3990(1)	1583(5)	69(1)
C (13)	5730(5)	4978(1)	2682(6)	81(2)
C (14)	5787(5)	5343(1)	3001(5)	67(1)
C (15)	5442(5)	5574(1)	1986(5)	79(2)
C (16)	5476(6)	5909(1)	2254(5)	83(2)
C (17)	5878(5)	6014(1)	3582(6)	81(2)

C (18)	6218(6)	5789(1)	4640(6)	89(2)
C (19)	6173(6)	5455(1)	4343(5)	83(2)
C (20)	6837(10)	6535(2)	4214(9)	114(2)
Cl (2)	9867(2)	6754(1)	7554(1)	93(1)
N (2)	13305(4)	6489(1)	10204(4)	69(1)
C (21)	13758(6)	6787(1)	10696(5)	75(1)
C (22)	13104(6)	7082(1)	10263(5)	70(1)
C (23)	11907(6)	7066(1)	9264(5)	73(1)
C (24)	11423(5)	6761(1)	8742(4)	64(1)
C (25)	12133(5)	6469(1)	9228(4)	58(1)
C (26)	13702(10)	7402(2)	10877(8)	96(2)
C (27)	11706(5)	6125(1)	8785(4)	58(1)
C (28)	11914(5)	5872(1)	9748(5)	66(1)
C (29)	11565(5)	5550(1)	9412(5)	73(1)
C (30)	10983(5)	5466(1)	8070(5)	69(1)
C (31)	10800(6)	5713(1)	7082(6)	74(1)
C (32)	11143(5)	6038(1)	7431(5)	68(1)
C (34)	9645(8)	4652(2)	6979(9)	125(3)
C (35)	10470(6)	4384(2)	7293(7)	108(2)
C (36)	9967(8)	4077(2)	7152(8)	117(2)
C (37)	8580(8)	4043(2)	6652(8)	114(2)
C (38)	7746(6)	4312(2)	6333(6)	99(2)
C (39)	8312(7)	4610(2)	6559(7)	107(2)
C (40)	7898(10)	3493(2)	6004(8)	98(2)

F (7)	12759(19)	7644(4)	10600(30)	176(8)
F (9)	14540(20)	7536(5)	10280(30)	175(12)
F (11)	14100(50)	7401(4)	12163(16)	259(16)
F (13)	7101(10)	3605(3)	4983(9)	155(4)
F (14)	7470(15)	3224(2)	6466(11)	144(4)
F (15)	9019(15)	3414(2)	5560(14)	120(5)
O (3)	10665(11)	5135(3)	8003(8)	66(2)
O (6)	8186(9)	3716(2)	7073(7)	105(3)
C (33)	10034(12)	5026(2)	6625(11)	75(3)
F (8)	14020(60)	7589(6)	9920(30)	220(20)
F (10)	14881(15)	7369(4)	11730(20)	114(7)
F (12)	12910(20)	7551(7)	11550(30)	147(10)
F (16)	6825(12)	3304(3)	5308(16)	157(5)
F (17)	7955(13)	3447(4)	7224(10)	149(5)
F (18)	8970(30)	3377(8)	5840(30)	253(18)
O (4)	7633(14)	3791(3)	5508(12)	116(4)
O (5)	10498(16)	5166(4)	7327(12)	77(4)
C (41)	10597(13)	4900(4)	8248(13)	78(4)

Table S4. Hydrogen coordinates (× 10^4) and isotropic displacement parameters (Å^2 × 10^3)

Atom	*x*	*y*	*z*	U_{eq}
H (1)	467	3247	-1520	110
H (3)	3439	2761	1004	88
H (8)	1388	4089	937	87

H (9)	1995	4622	1561	90
H (11)	5993	4369	2205	85
H (12)	5349	3830	1598	83
H (13A)	6175	4935	1923	97
H (13B)	6215	4854	3472	97
H (15)	5175	5503	1083	94
H (16)	5230	6062	1546	99
H (18)	6475	5862	5542	106
H (19)	6404	5301	5052	99
H (21)	14565	6796	11372	90
H (23)	11431	7260	8945	87
H (28)	12307	5923	10656	79
H (29)	11717	5387	10086	88
H (32)	10998	6201	6756	81
H (35)	11415	4416	7616	130
H (36)	10536	3893	7384	140
H (38)	6805	4287	5967	119
H (39)	7742	4796	6414	128
H (33A)	10683	5031	6021	90
H (33B)	9220	5156	6229	90
H (41A)	10143	4942	9002	94
H (41B)	11536	4823	8583	94
H (31)	10410(50)	5662(11)	6210(50)	81(16)

Table S5. Bond lengths of compound 7a

Atoms	Length (Å)	Atoms	Length (Å)
Cl(1)-C(4)	1.729(5)	F(1)-C(6)	1.233(8)
F(2)-C(6)	1.246(9)	F(3)-C(6)	1.234(8)
F(4)-C(20)	1.255(9)	F(5)-C(20)	1.195(9)
F(6)-C(20)	1.258(8)	O(1)-C(10)	1.367(5)
O(1)-C(13)	1.420(6)	O(2)-C(17)	1.415(6)
O(2)-C(20)	1.249(8)	N(1)-C(1)	1.329(6)
N(1)-C(5)	1.354(6)	C(1)-H(1)	0.93
C(1)-C(2)	1.367(7)	C(2)-C(3)	1.371(7)
C(2)-C(6)	1.462(8)	C(3)-H(3)	0.93
C(3)-C(4)	1.373(6)	C(4)-C(5)	1.408(6)
C(5)-C(7)	1.478(6)	C(7)-C(8)	1.387(6)
C(7)-C(12)	1.374(6)	C(8)-H(8)	0.93
C(8)-C(9)	1.361(6)	C(9)-H(9)	0.93
C(9)-C(10)	1.386(6)	C(10)-C(11)	1.379(6)
C(11)-H(11)	0.93	C(11)-C(12)	1.384(6)
C(12)-H(12)	0.93	C(13)-H(13A)	0.97
C(13)-H(13B)	0.97	C(13)-C(14)	1.500(6)
C(14)-C(15)	1.364(6)	C(14)-C(19)	1.388(7)
C(15)-H(15)	0.93	C(15)-C(16)	1.372(7)
C(16)-H(16)	0.93	C(16)-C(17)	1.367(7)
C(17)-C(18)	1.377(7)	C(18)-H(18)	0.93
C(18)-C(19)	1.374(7)	C(19)-H(19)	0.93
Cl(2)-C(24)	1.733(5)	N(2)-C(21)	1.336(6)

N(2)-C(25)	1.349(6)	C(21)-H(21)	0.93
C(21)-C(22)	1.374(6)	C(22)-C(23)	1.378(7)
C(22)-C(26)	1.494(7)	C(23)-H(23)	0.93
C(23)-C(24)	1.379(6)	C(24)-C(25)	1.399(6)
C(25)-C(27)	1.485(6)	C(26)-F(7)	1.334(17)
C(26)-F(9)	1.238(16)	C(26)-F(11)	1.261(15)
C(26)-F(8)	1.31(3)	C(26)-F(10)	1.295(15)
C(26)-F(12)	1.283(18)	C(27)-C(28)	1.387(6)
C(27)-C(32)	1.393(6)	C(28)-H(28)	0.93
C(28)-C(29)	1.361(6)	C(29)-H(29)	0.93
C(29)-C(30)	1.384(7)	C(30)-C(31)	1.384(7)
C(30)-O(3)	1.363(11)	C(30)-O(5)	1.442(16)
C(31)-C(32)	1.377(6)	C(31)-H(31)	0.90(5)
C(32)-H(32)	0.93	C(34)-C(35)	1.347(9)
C(34)-C(39)	1.307(9)	C(34)-C(33)	1.611(12)
C(34)-C(41)	1.727(15)	C(35)-H(35)	0.93
C(35)-C(36)	1.329(9)	C(36)-H(36)	0.93
C(36)-C(37)	1.363(9)	C(37)-C(38)	1.356(9)
C(37)-O(6)	1.461(10)	C(37)-O(4)	1.661(14)
C(38)-H(38)	0.93	C(38)-C(39)	1.321(8)
C(39)-H(39)	0.93	C(40)-F(13)	1.235(11)
C(40)-F(14)	1.282(10)	C(40)-F(15)	1.319(15)
C(40)-O(6)	1.376(10)	C(40)-F(16)	1.368(12)
C(40)-F(17)	1.221(12)	C(40)-F(18)	1.20(3)

C(40)-O(4)	1.300(12)	O(3)-C(33)	1.453(13)
C(33)-H(33A)	0.97	C(33)-H(33B)	0.97
O(5)-C(41)	1.400(18)	C(41)-H(41A)	0.97
C(41)-H(41B)	0.97		

Table S6. Torsion angles of compound 7a

Atoms	Angle (°)	Atoms	Angle (°)
C(5)-N(1)-C(1)-C(2)	-1.0(9)	N(1)-C(1)-C(2)-C(3)	2.1(9)
N(1)-C(1)-C(2)-C(6)	-179.0(7)	C(1)-C(2)-C(3)-C(4)	-1.1(8)
C(6)-C(2)-C(3)-C(4)	-180.0(6)	C(2)-C(3)-C(4)-C(5)	-0.9(8)
C(2)-C(3)-C(4)-Cl(1)	176.7(4)	C(1)-N(1)-C(5)-C(4)	-1.0(8)
C(1)-N(1)-C(5)-C(7)	179.6(5)	C(3)-C(4)-C(5)-N(1)	2.0(7)
Cl(1)-C(4)-C(5)-N(1)	-175.5(4)	C(3)-C(4)-C(5)-C(7)	-178.7(5)
Cl(1)-C(4)-C(5)-C(7)	3.8(7)	C(1)-C(2)-C(6)-F(1)	-2.2(13)
C(3)-C(2)-C(6)-F(1)	176.6(8)	C(1)-C(2)-C(6)-F(3)	-124.1(9)
C(3)-C(2)-C(6)-F(3)	54.7(12)	C(1)-C(2)-C(6)-F(2)	118.5(9)
C(3)-C(2)-C(6)-F(2)	-62.7(11)	N(1)-C(5)-C(7)-C(12)	-142.9(5)
C(4)-C(5)-C(7)-C(12)	37.8(7)	N(1)-C(5)-C(7)-C(8)	34.2(6)
C(4)-C(5)-C(7)-C(8)	-145.1(5)	C(12)-C(7)-C(8)-C(9)	0.3(7)
C(5)-C(7)-C(8)-C(9)	-177.0(5)	C(7)-C(8)-C(9)-C(10)	-0.3(8)
C(13)-O(1)-C(10)-C(11)	2.5(7)	C(13)-O(1)-C(10)-C(9)	-177.1(4)
C(8)-C(9)-C(10)-O(1)	-179.7(4)	C(8)-C(9)-C(10)-C(11)	0.7(7)
O(1)-C(10)-C(11)-C(12)	179.2(4)	C(9)-C(10)-C(11)-C(12)	-1.2(7)
C(8)-C(7)-C(12)-C(11)	-0.8(7)	C(5)-C(7)-C(12)-C(11)	176.4(4)

C(10)-C(11)-C(12)-C(7)	1.3(8)	C(10)-O(1)-C(13)-C(14)	178.0(4)
O(1)-C(13)-C(14)-C(15)	-73.5(6)	O(1)-C(13)-C(14)-C(19)	105.5(6)
C(19)-C(14)-C(15)-C(16)	0.5(8)	C(13)-C(14)-C(15)-C(16)	179.5(5)
C(14)-C(15)-C(16)-C(17)	0.4(8)	C(15)-C(16)-C(17)-C(18)	-1.3(9)
C(15)-C(16)-C(17)-O(2)	-174.2(5)	C(20)-O(2)-C(17)-C(16)	-109.3(8)
C(20)-O(2)-C(17)-C(18)	77.7(9)	C(16)-C(17)-C(18)-C(19)	1.1(9)
O(2)-C(17)-C(18)-C(19)	173.9(5)	C(17)-C(18)-C(19)-C(14)	-0.2(9)
C(15)-C(14)-C(19)-C(18)	-0.6(8)	C(13)-C(14)-C(19)-C(18)	-179.6(5)
C(17)-O(2)-C(20)-F(5)	-55.4(12)	C(17)-O(2)-C(20)-F(4)	59.4(11)
C(17)-O(2)-C(20)-F(6)	173.2(6)	C(25)-N(2)-C(21)-C(22)	-0.4(8)
N(2)-C(21)-C(22)-C(23)	0.2(8)	N(2)-C(21)-C(22)-C(26)	-179.7(6)
C(21)-C(22)-C(23)-C(24)	-0.3(7)	C(26)-C(22)-C(23)-C(24)	179.7(6)
C(22)-C(23)-C(24)-C(25)	0.5(7)	C(22)-C(23)-C(24)-Cl(2)	176.7(4)
C(21)-N(2)-C(25)-C(24)	0.7(7)	C(21)-N(2)-C(25)-C(27)	-178.4(4)
C(23)-C(24)-C(25)-N(2)	-0.7(7)	Cl(2)-C(24)-C(25)-N(2)	-176.7(3)
C(23)-C(24)-C(25)-C(27)	178.2(4)	Cl(2)-C(24)-C(25)-C(27)	2.3(7)
C(21)-C(22)-C(26)-F(9)	89.8(18)	C(23)-C(22)-C(26)-F(9)	-90.2(19)
C(21)-C(22)-C(26)-F(11)	-45(3)	C(23)-C(22)-C(26)-F(11)	135(3)
C(21)-C(22)-C(26)-F(12)	-115.5(19)	C(23)-C(22)-C(26)-F(12)	65(2)
C(21)-C(22)-C(26)-F(10)	4.8(15)	C(23)-C(22)-C(26)-F(10)	-175.2(13)
C(21)-C(22)-C(26)-F(8)	118(3)	C(23)-C(22)-C(26)-F(8)	-62(3)
C(21)-C(22)-C(26)-F(7)	-163.8(18)	C(23)-C(22)-C(26)-F(7)	16(2)
N(2)-C(25)-C(27)-C(28)	36.6(6)	C(24)-C(25)-C(27)-C(28)	-142.4(5)
N(2)-C(25)-C(27)-C(32)	-141.2(5)	C(24)-C(25)-C(27)-C(32)	39.8(7)

C(32)-C(27)-C(28)-C(29)	-1.3(7)	C(25)-C(27)-C(28)-C(29)	-179.2(4)
C(27)-C(28)-C(29)-C(30)	0.2(8)	C(28)-C(29)-C(30)-O(3)	-177.1(6)
C(28)-C(29)-C(30)-C(31)	1.6(8)	C(28)-C(29)-C(30)-O(5)	179.5(10)
O(3)-C(30)-C(31)-C(32)	176.0(8)	C(29)-C(30)-C(31)-C(32)	-2.2(8)
O(5)-C(30)-C(31)-C(32)	179.2(8)	C(30)-C(31)-C(32)-C(27)	1.1(8)
C(28)-C(27)-C(32)-C(31)	0.6(7)	C(25)-C(27)-C(32)-C(31)	178.4(5)
C(39)-C(34)-C(35)-C(36)	1.8(12)	C(33)-C(34)-C(35)-C(36)	-159.1(9)
C(41)-C(34)-C(35)-C(36)	146.3(8)	C(34)-C(35)-C(36)-C(37)	1.1(12)
C(35)-C(36)-C(37)-C(38)	-1.0(12)	C(35)-C(36)-C(37)-O(6)	-158.5(7)
C(35)-C(36)-C(37)-O(4)	136.9(9)	C(36)-C(37)-C(38)-C(39)	-1.9(11)
O(6)-C(37)-C(38)-C(39)	150.8(8)	O(4)-C(37)-C(38)-C(39)	-152.7(7)
C(35)-C(34)-C(39)-C(38)	-5.0(12)	C(33)-C(34)-C(39)-C(38)	159.5(7)
C(41)-C(34)-C(39)-C(38)	-134.1(11)	C(37)-C(38)-C(39)-C(34)	5.0(12)
C(31)-C(30)-O(3)-C(33)	1.0(14)	C(29)-C(30)-O(3)-C(33)	179.4(8)
F(13)-C(40)-O(6)-C(37)	-48.8(12)	F(14)-C(40)-O(6)-C(37)	-175.4(11)
F(15)-C(40)-O(6)-C(37)	68.3(11)	C(38)-C(37)-O(6)-C(40)	96.9(10)
C(36)-C(37)-O(6)-C(40)	-107.2(9)	C(30)-O(3)-C(33)-C(34)	-173.3(7)
C(39)-C(34)-C(33)-O(3)	122.0(9)	C(35)-C(34)-C(33)-O(3)	-75.6(12)
F(18)-C(40)-O(4)-C(37)	-79.4(17)	F(17)-C(40)-O(4)-C(37)	42.9(14)
F(16)-C(40)-O(4)-C(37)	161.6(10)	C(38)-C(37)-O(4)-C(40)	-153.6(9)
C(36)-C(37)-O(4)-C(40)	61.4(13)	C(31)-C(30)-O(5)-C(41)	-178.0(10)
C(29)-C(30)-O(5)-C(41)	3.8(18)	C(30)-O(5)-C(41)-C(34)	169.3(9)
C(39)-C(34)-C(41)-O(5)	-86.8(13)	C(35)-C(34)-C(41)-O(5)	135.9(10)

Table S7. Hydrogen bond parameters of compound 7a

D-H···A	d(D-H) (Å)	d(H···A) (Å)	d(D···A) (Å)	<(DHA) (Å)
C(16)-H(16)···N(2)	0.93	2.7	3.516(6)	147.3

2. ^{1}H NMR, ^{13}C NMR, and HRMS spectra of target compounds 7a-7r

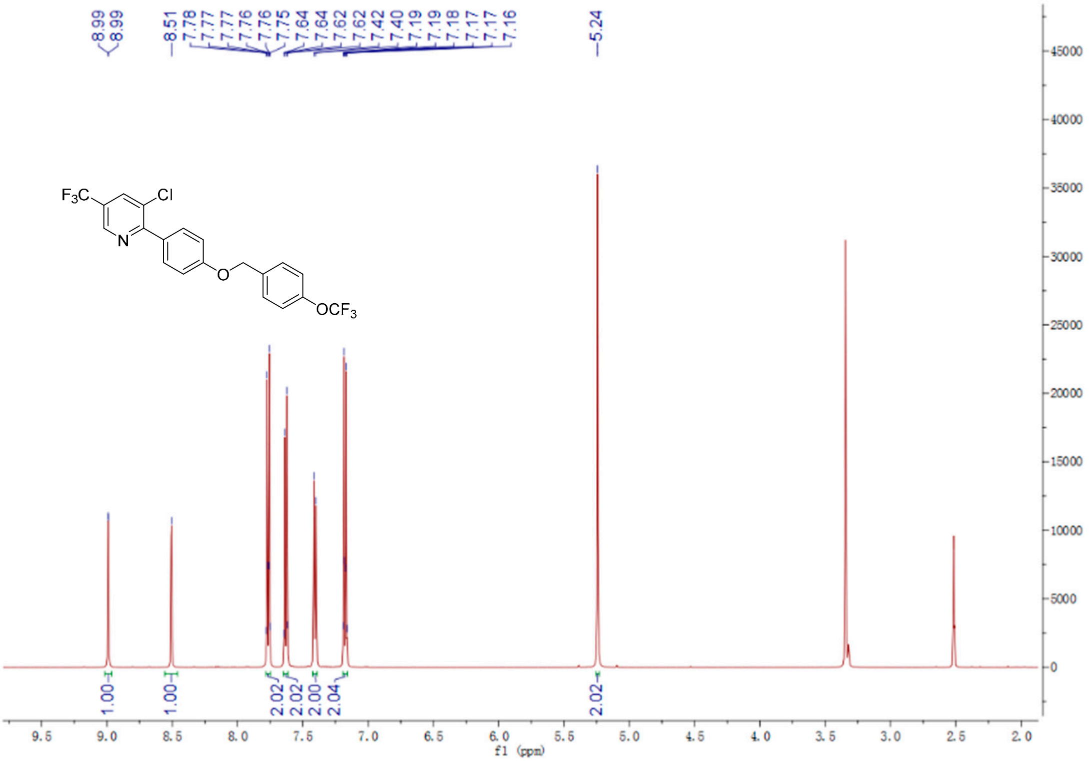

Figure S1. The ^{1}H NMR spectrum of **7a** (DMSO-*d*$_6$)

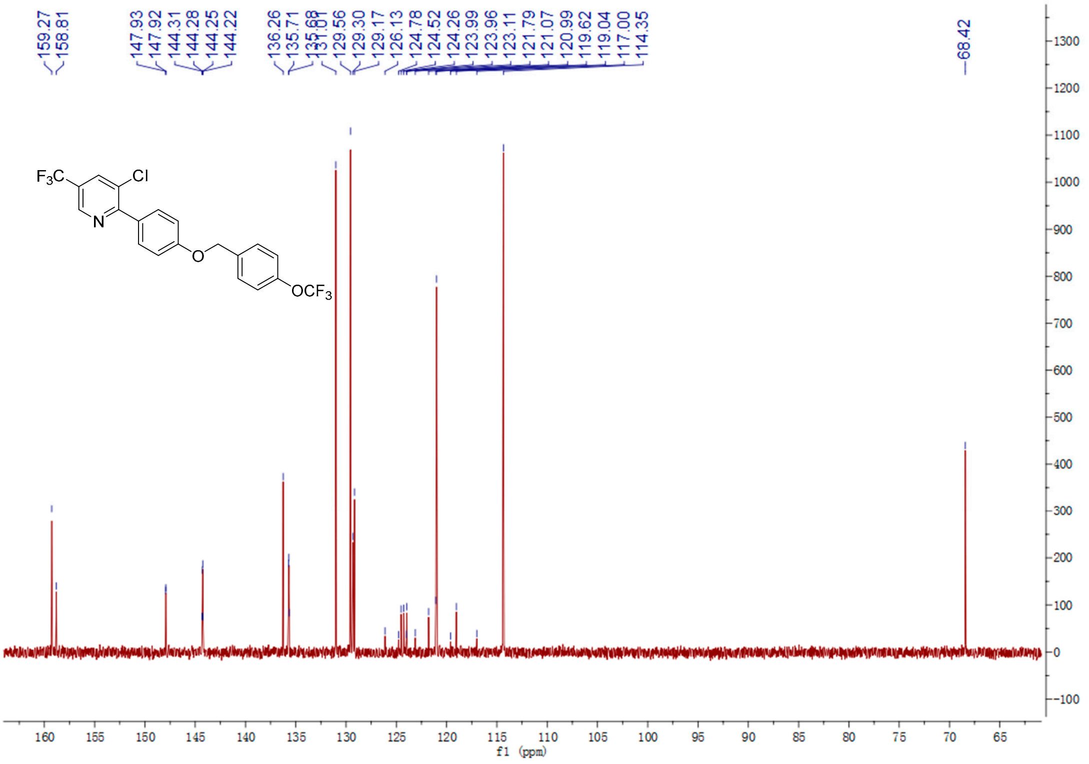

Figure S2. The ^{13}C NMR spectrum of **7a** (DMSO-*d*$_6$)

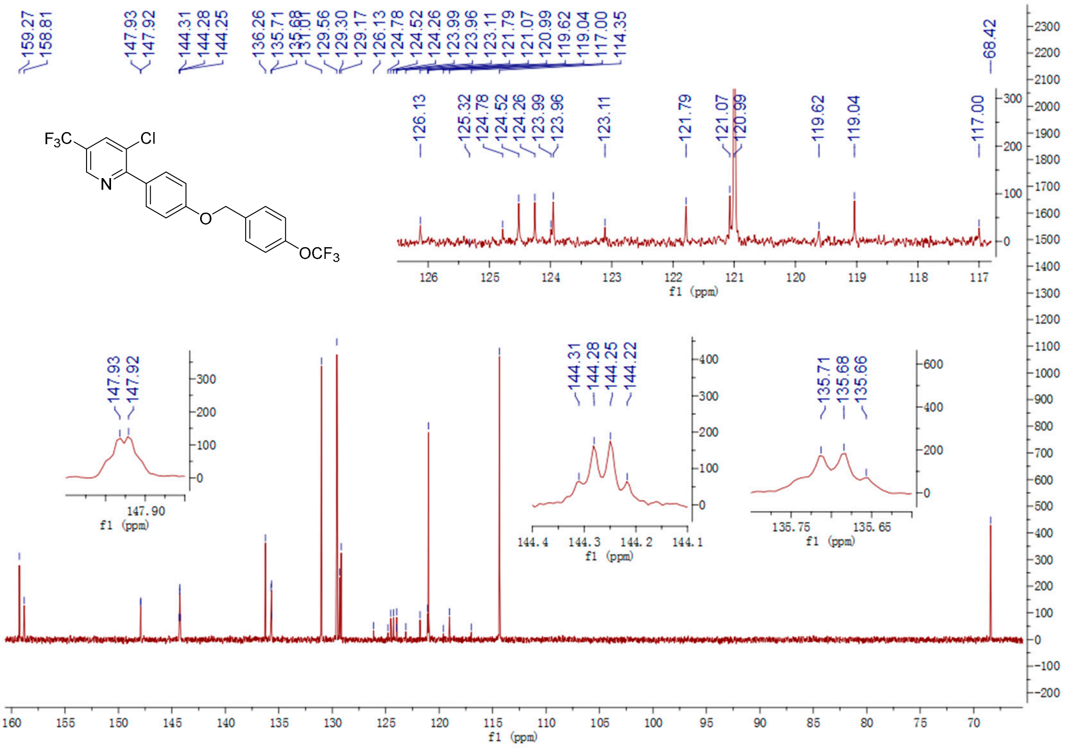

Figure S3. The ^{13}C NMR spectrum of **7a** (DMSO-d_6)

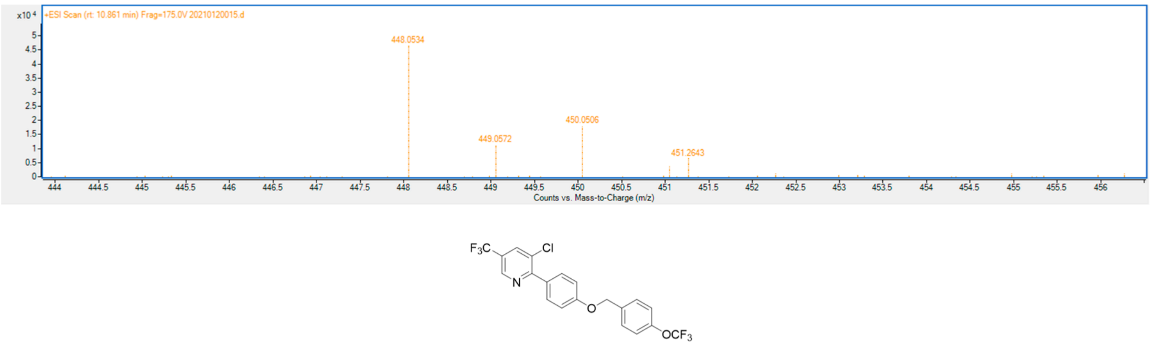

Figure S4. The HRMS spectrum of **7a**

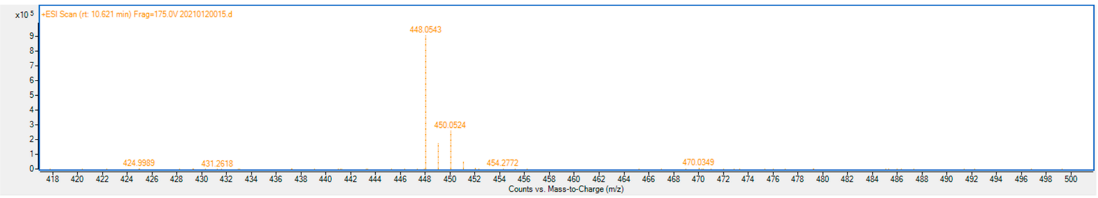

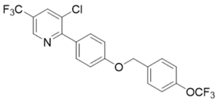

Figure S5. The HRMS spectrum of **7a**

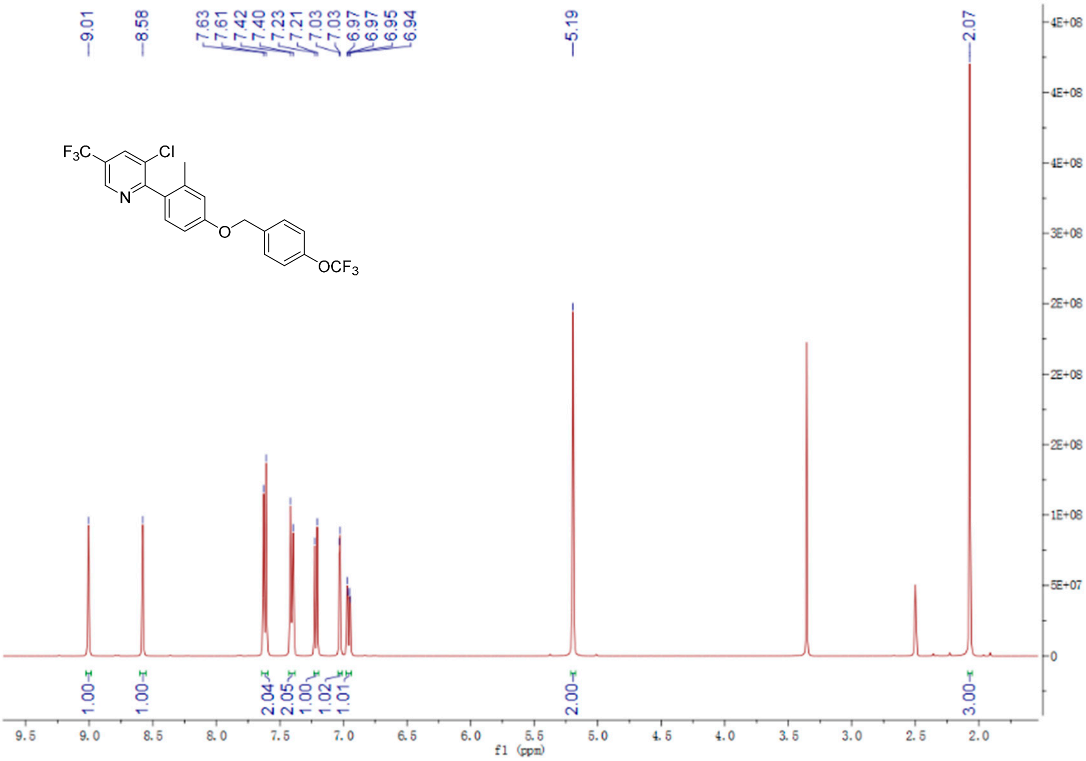

Figure S6. The ^{1}H NMR spectrum of **7b** (DMSO-d_6)

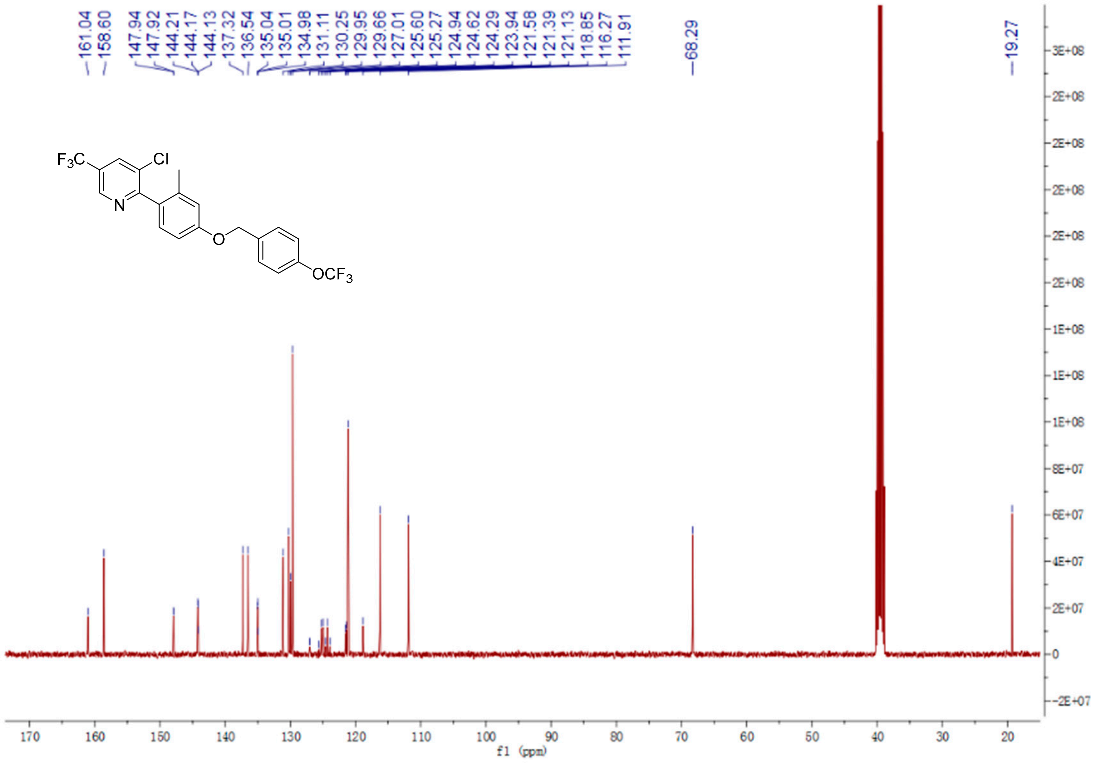

Figure S7. The ^{13}C NMR spectrum of **7b** (DMSO-*d*$_6$)

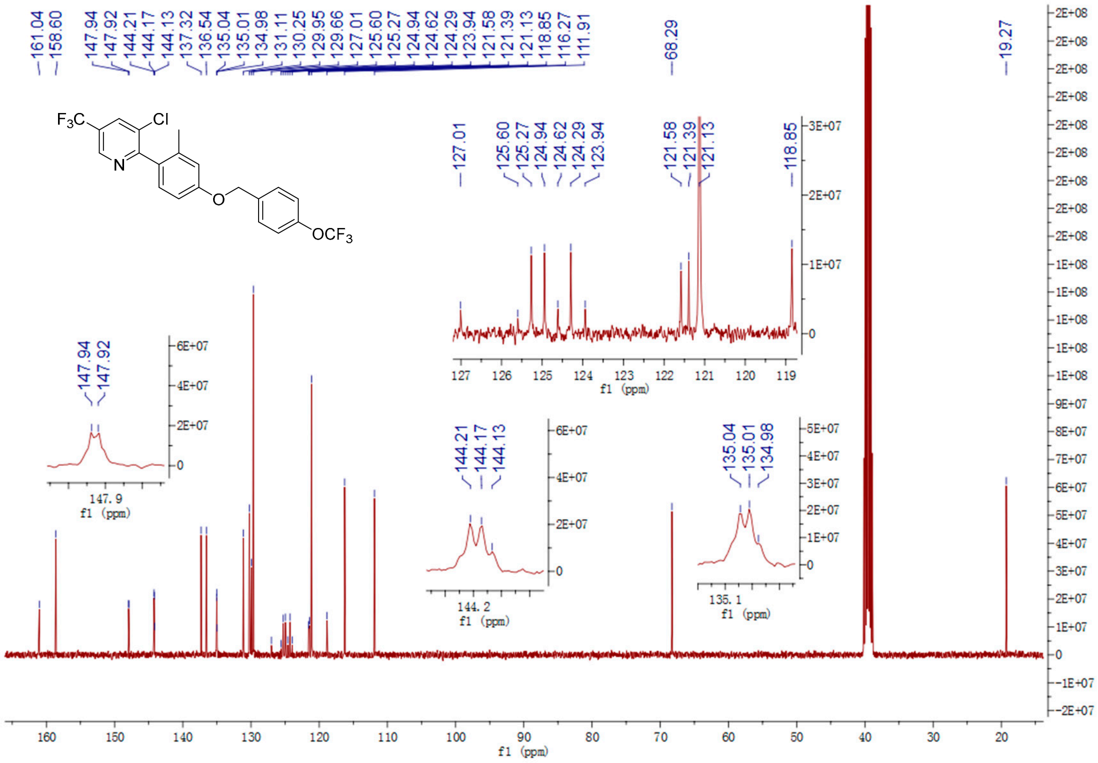

Figure S8. The ^{13}C NMR spectrum of **7b** (DMSO-*d*$_6$)

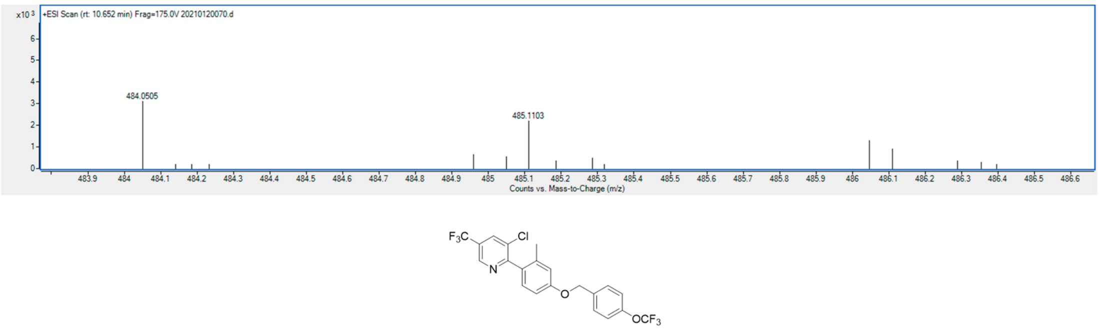

Figure S9. The HRMS spectrum of **7b**

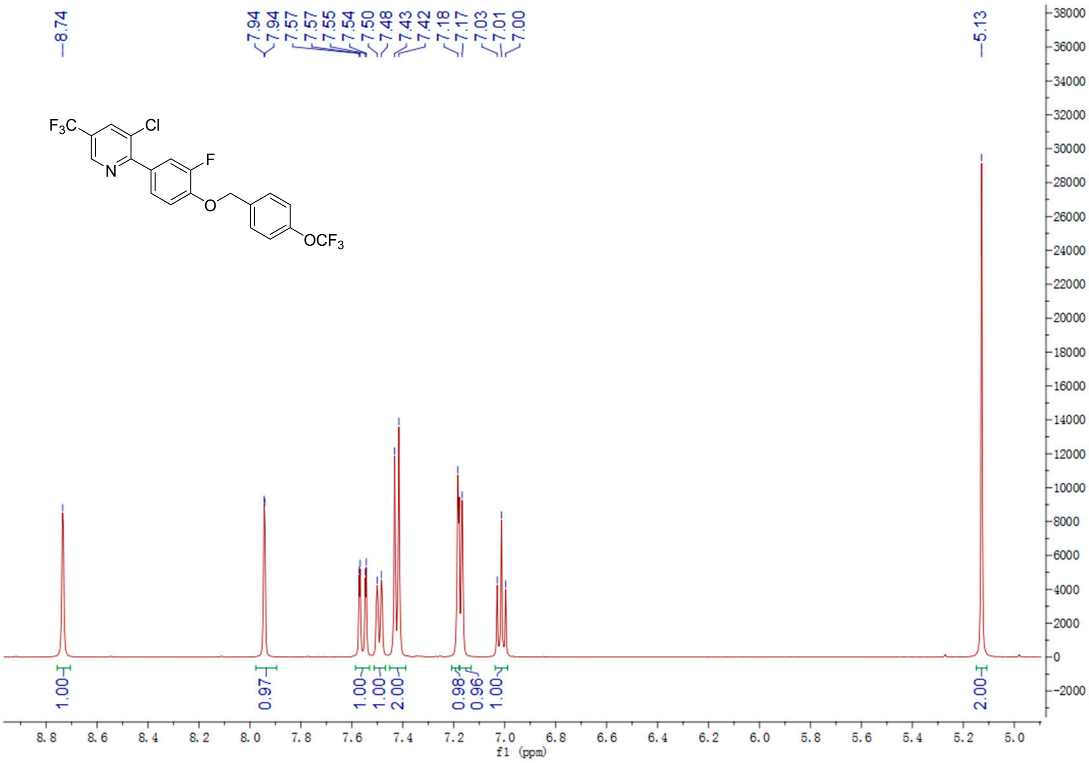

Figure S10. The ^{1}H NMR spectrum of **7c** ($CDCl_3$)

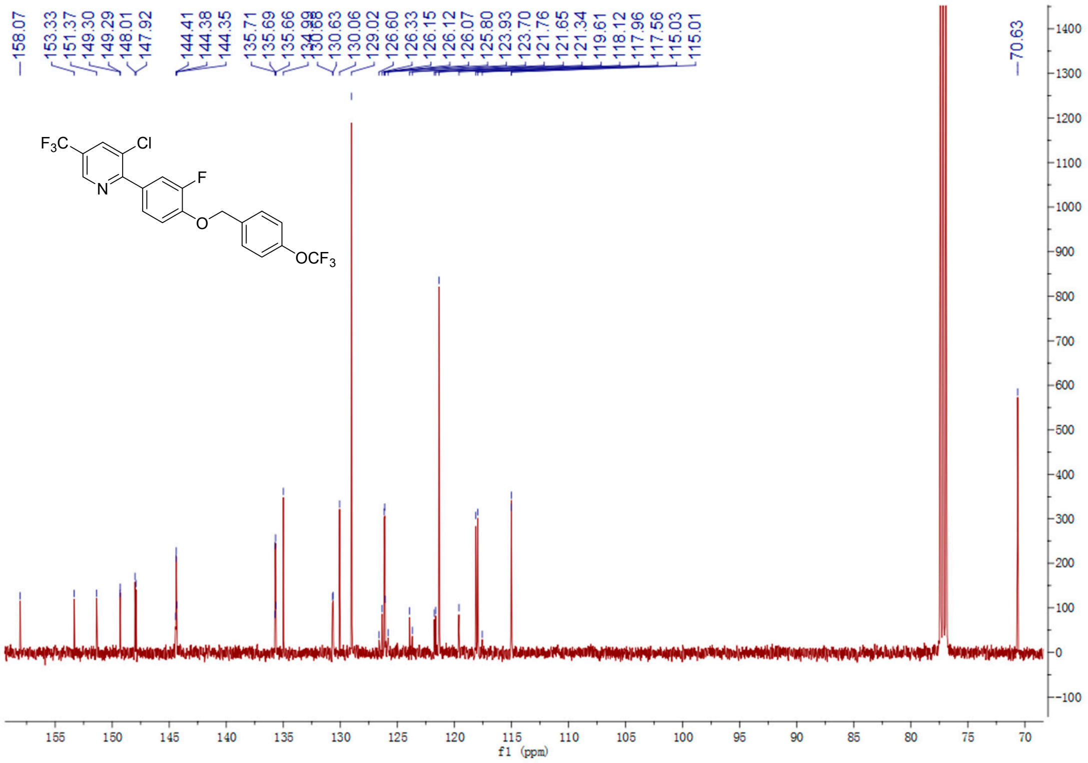

Figure S11. The ^{13}C NMR spectrum of **7c** ($CDCl_3$)

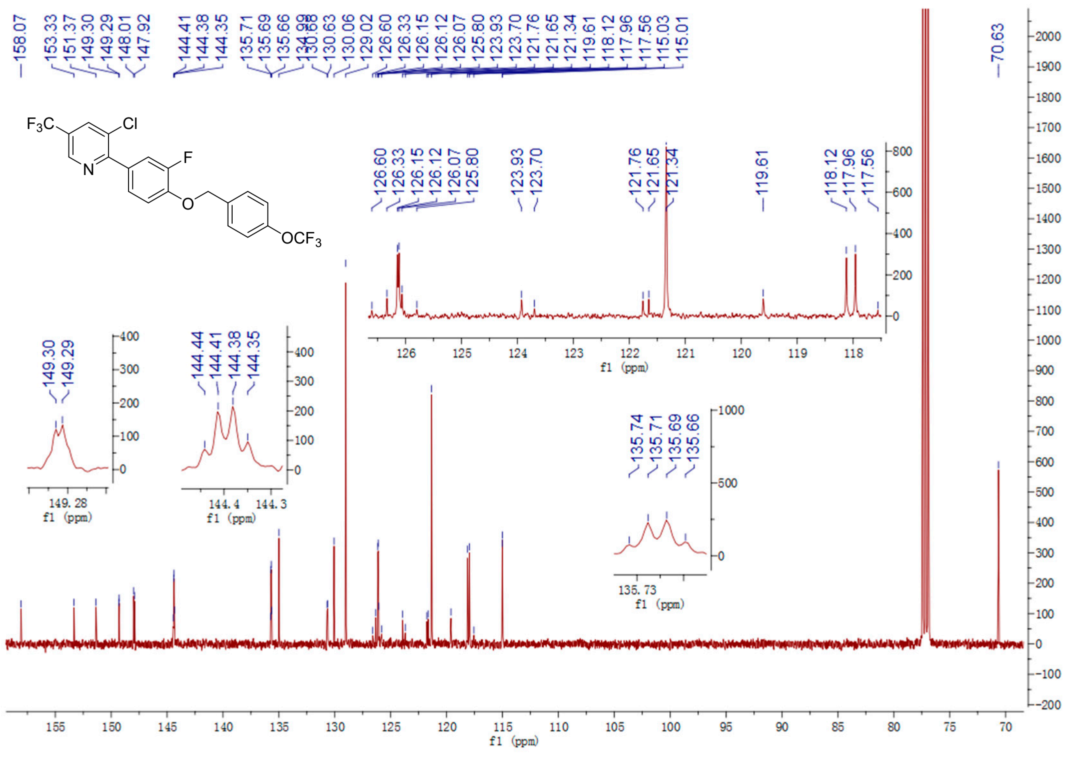

Figure S12. The ^{13}C NMR spectrum of **7c** ($CDCl_3$)

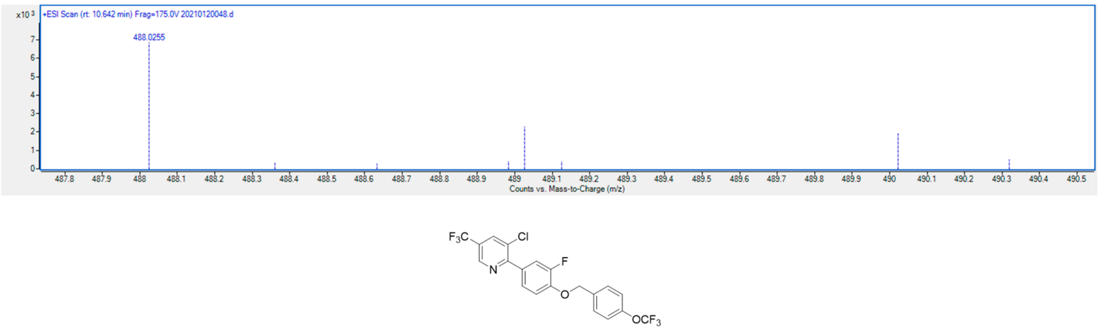

Figure S13. The HRMS spectrum of **7c**

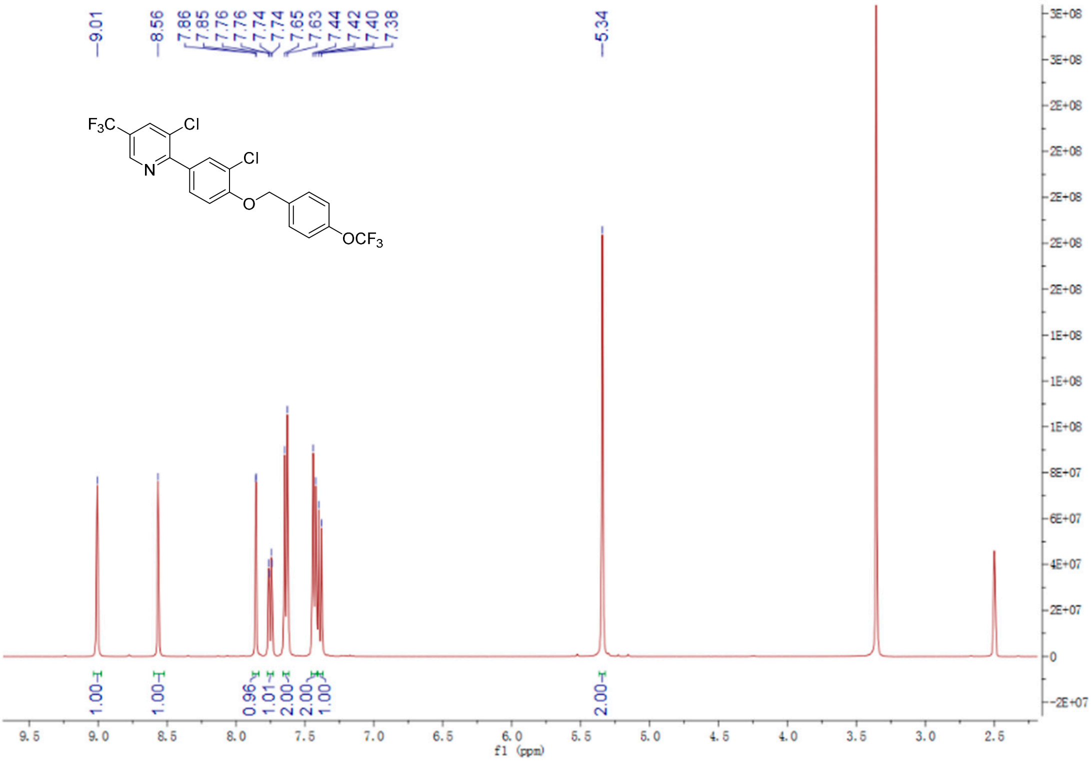

Figure S14. The ^{1}H NMR spectrum of **7d** (DMSO-d_6)

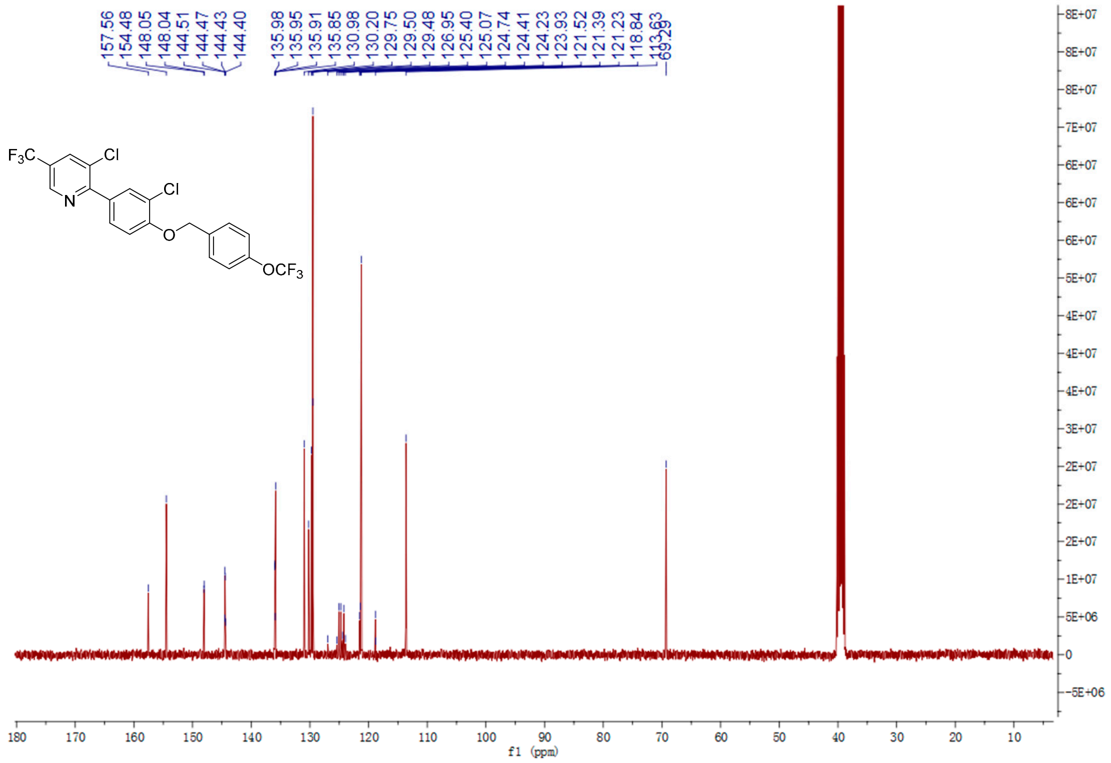

Figure S15. The ^{13}C NMR spectrum of **7d** (DMSO-*d*[6])

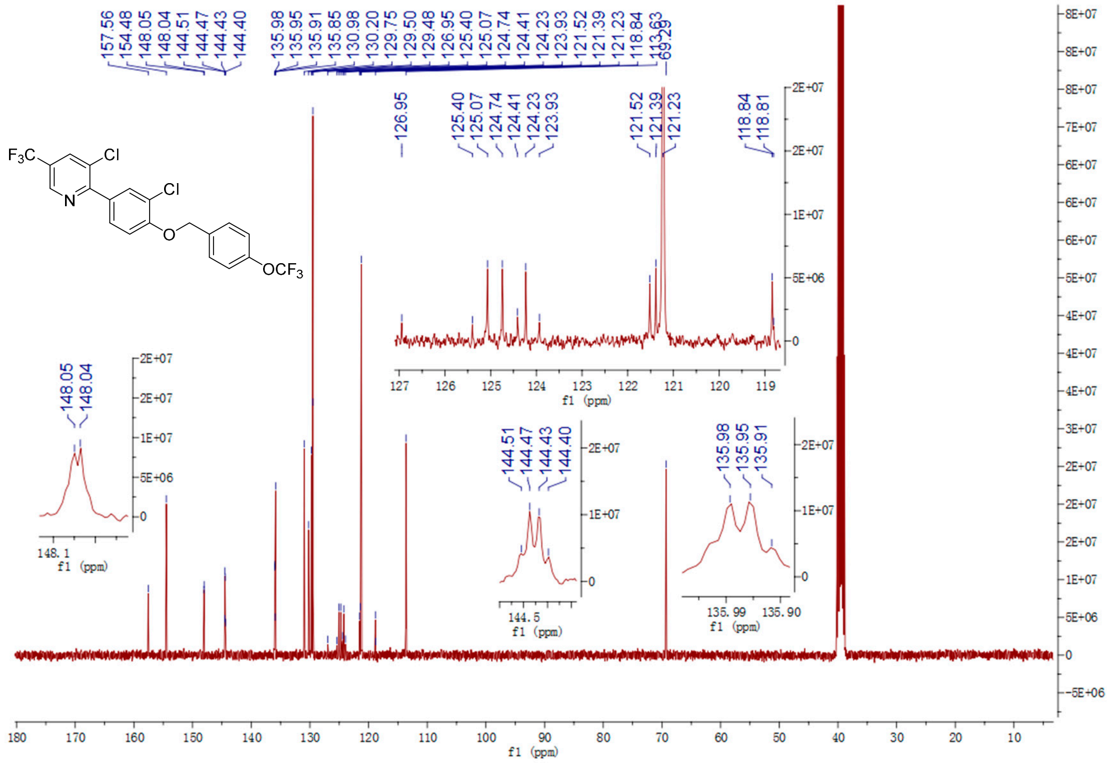

Figure S16. The ^{13}C NMR spectrum of **7d** (DMSO-*d*[6])

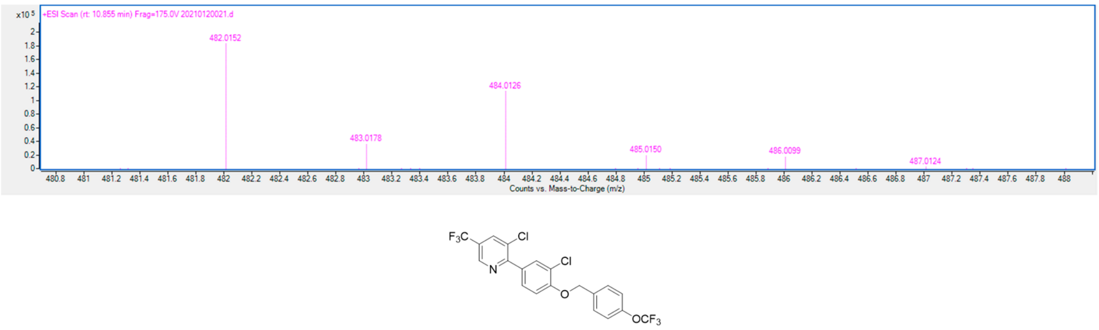

Figure S17. The HRMS spectrum of **7d**

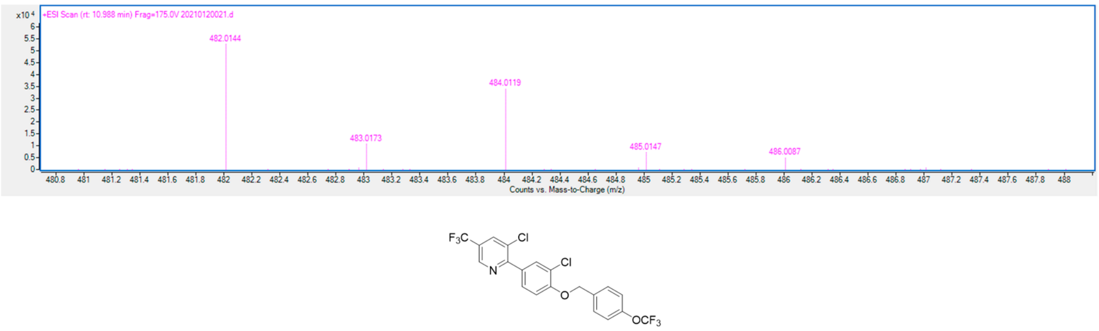

Figure S18. The HRMS spectrum of **7d**

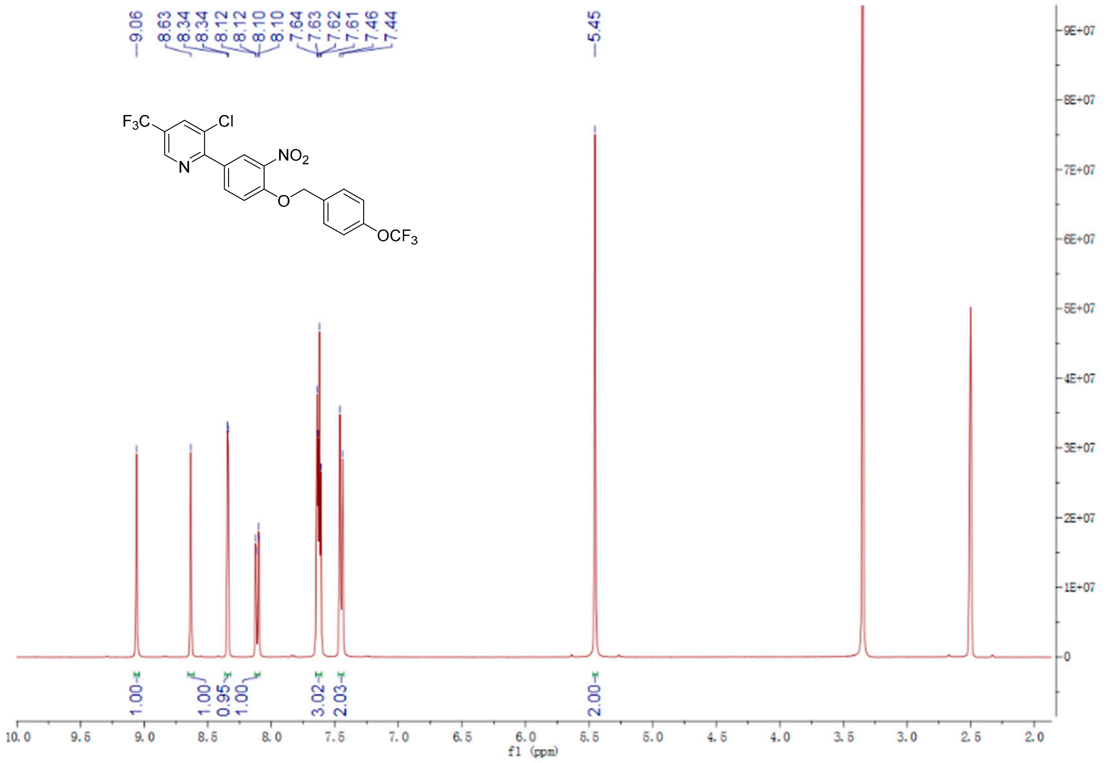

Figure S19. The ¹H NMR spectrum of **7e** (DMSO-d_6)

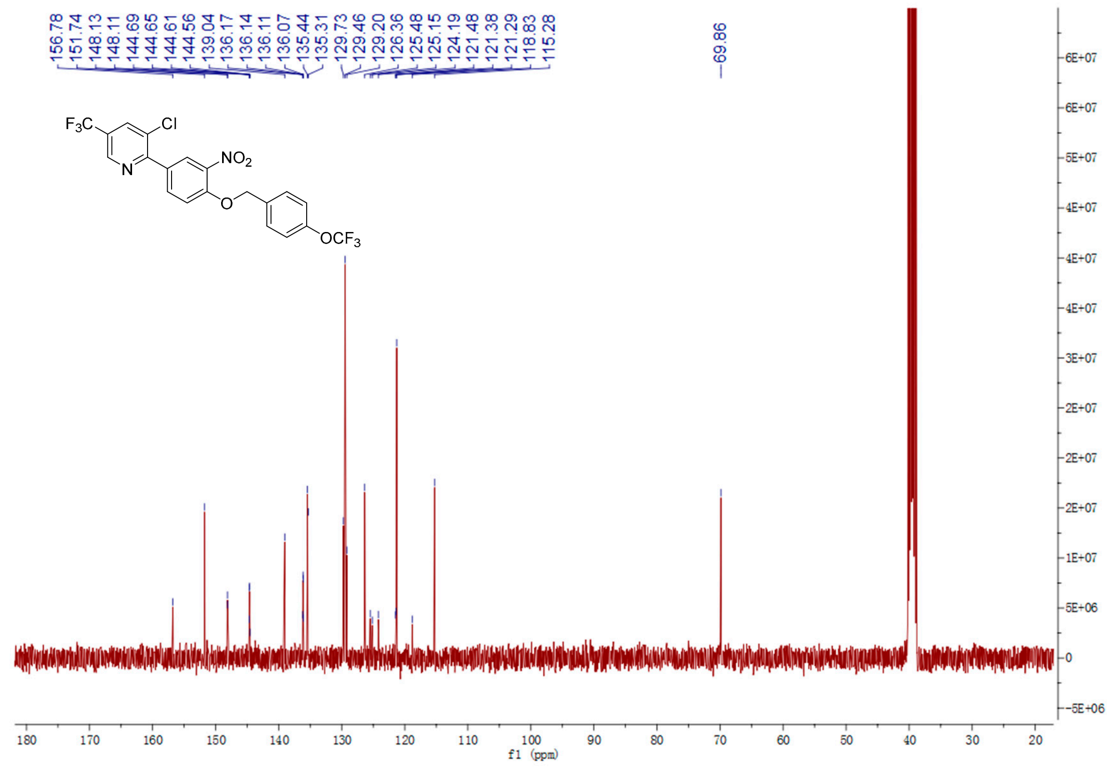

Figure S20. The ¹³C NMR spectrum of **7e** (DMSO-d_6)

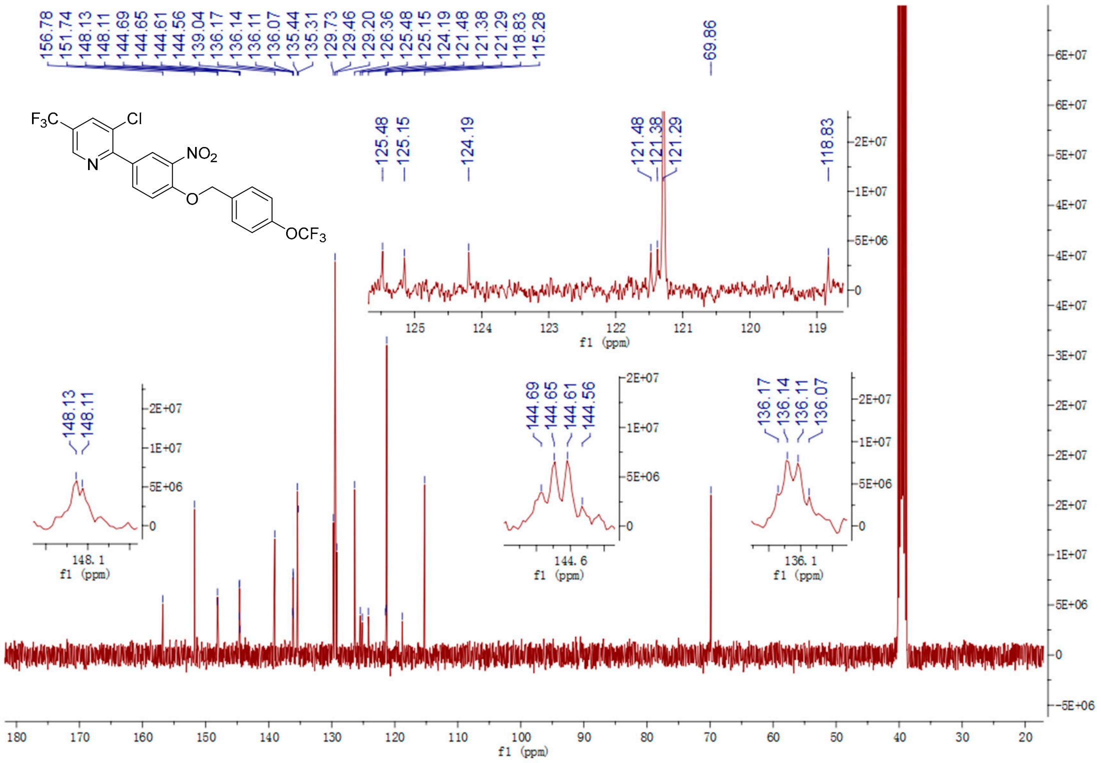

Figure S21. The ^{13}C NMR spectrum of **7e** (DMSO-d_6)

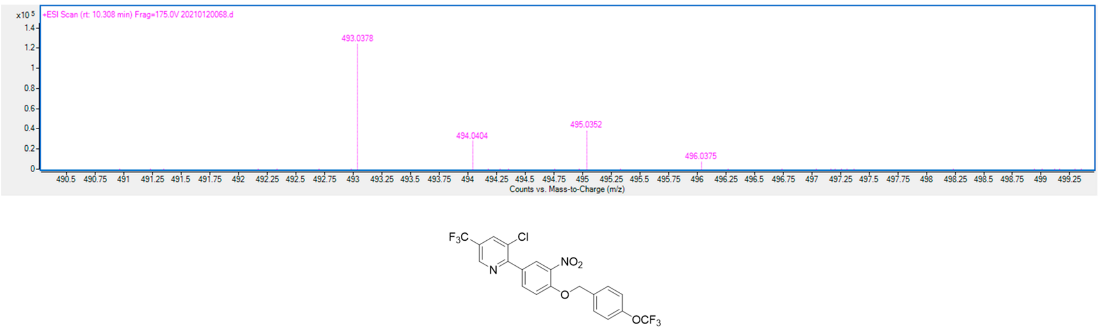

Figure S22. The HRMS spectrum of **7e**

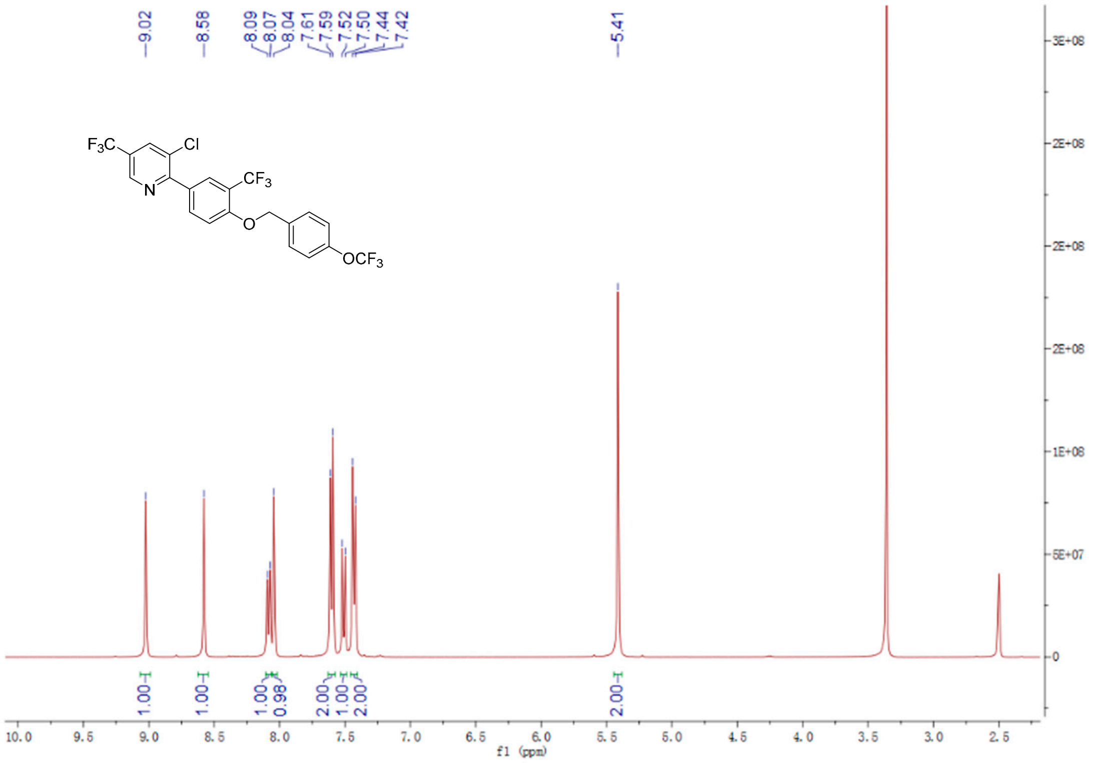

Figure S23. The ^{1}H NMR spectrum of **7f** (DMSO-*d6*)

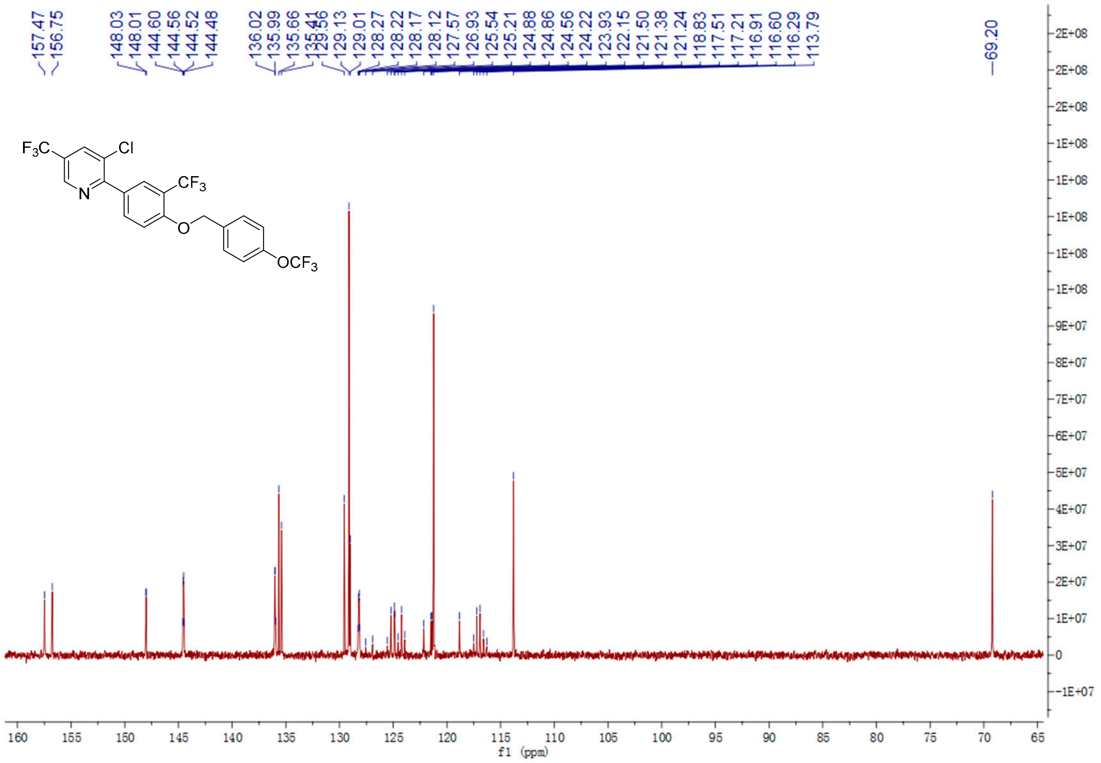

Figure S24. The ^{13}C NMR spectrum of **7f** (DMSO-*d6*)

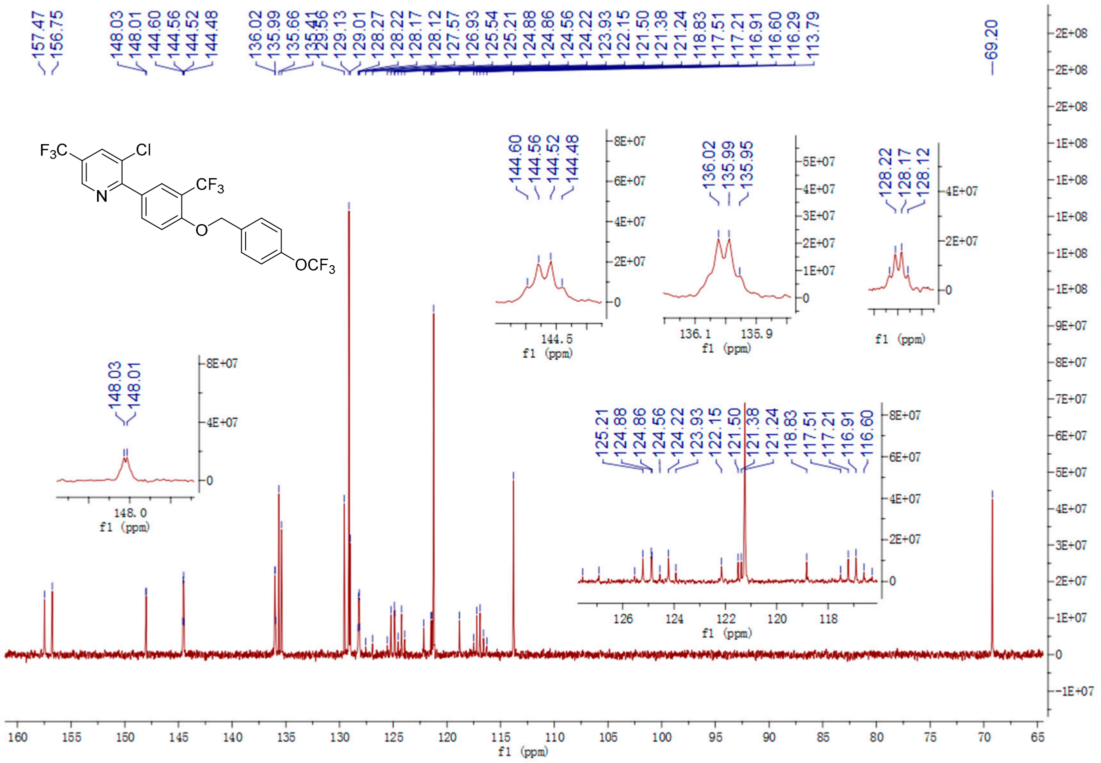

Figure S25. The ^{13}C NMR spectrum of **7f** (DMSO-d_6)

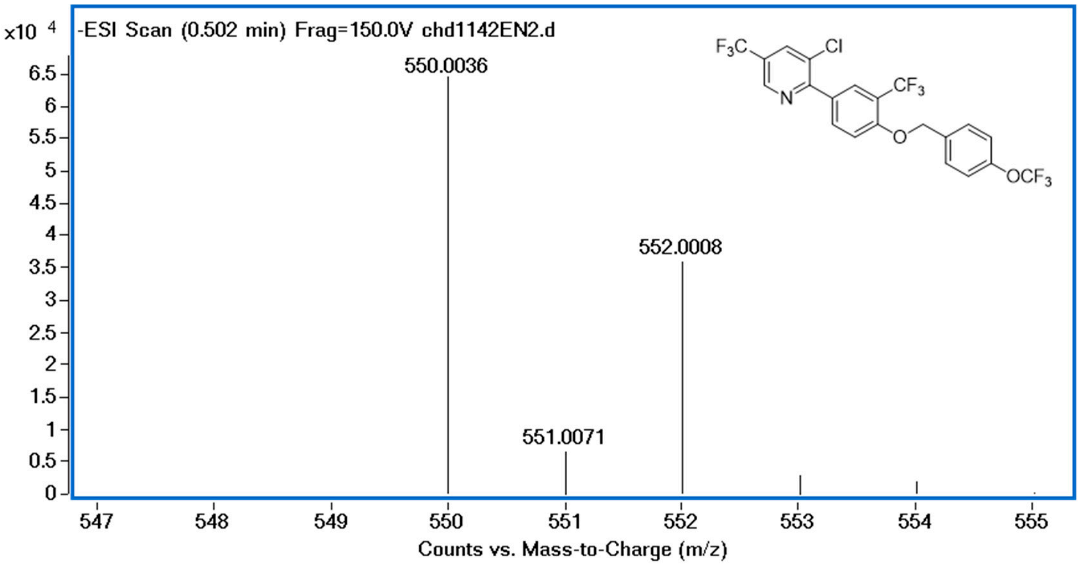

Figure S26. The HRMS spectrum of **7f**

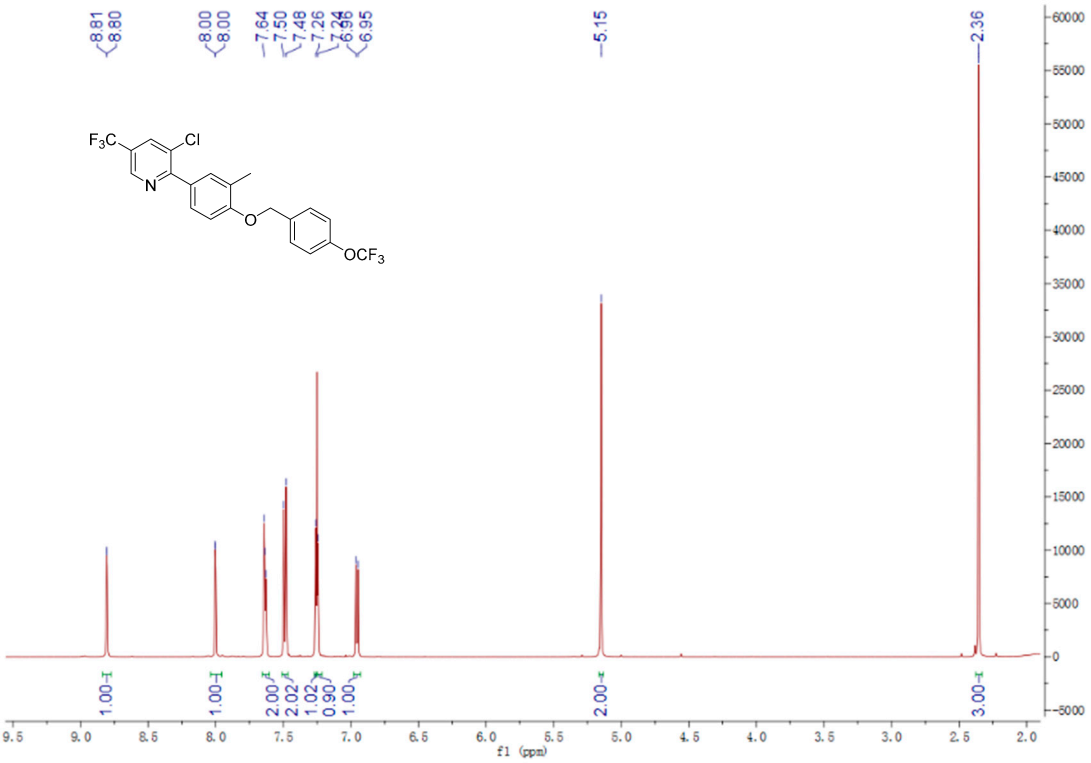

Figure S27. The ^{1}H NMR spectrum of **7g** ($CDCl_3$)

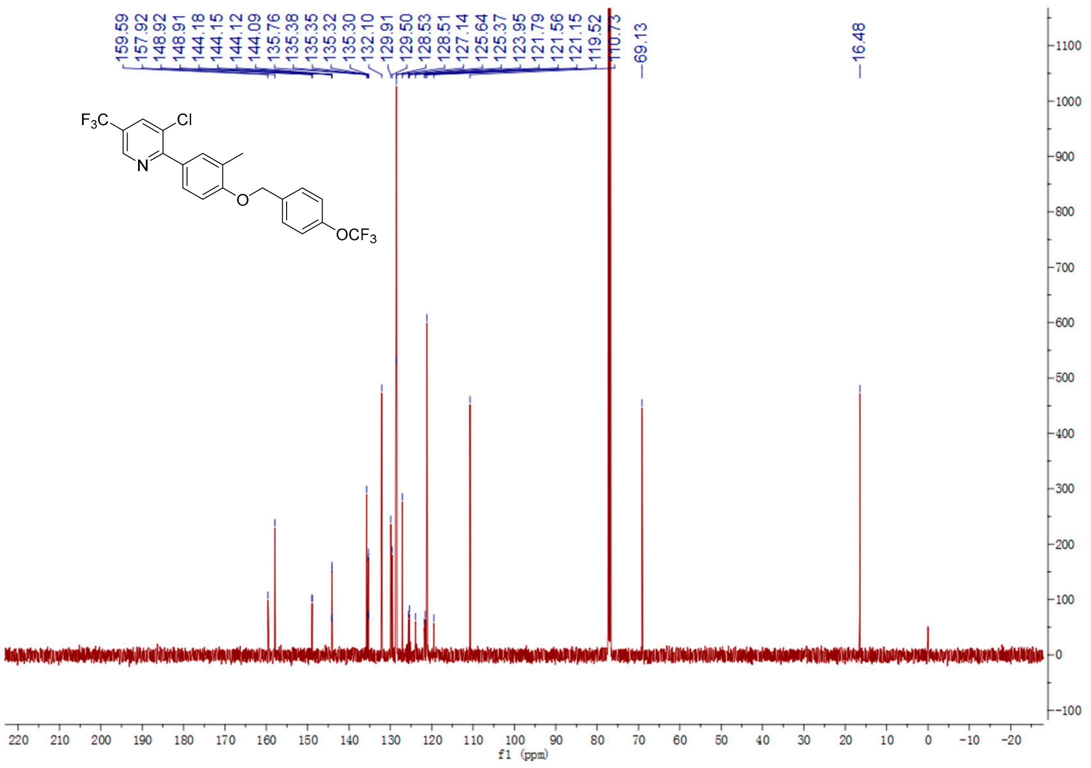

Figure S28. The ^{13}C NMR spectrum of **7g** ($CDCl_3$)

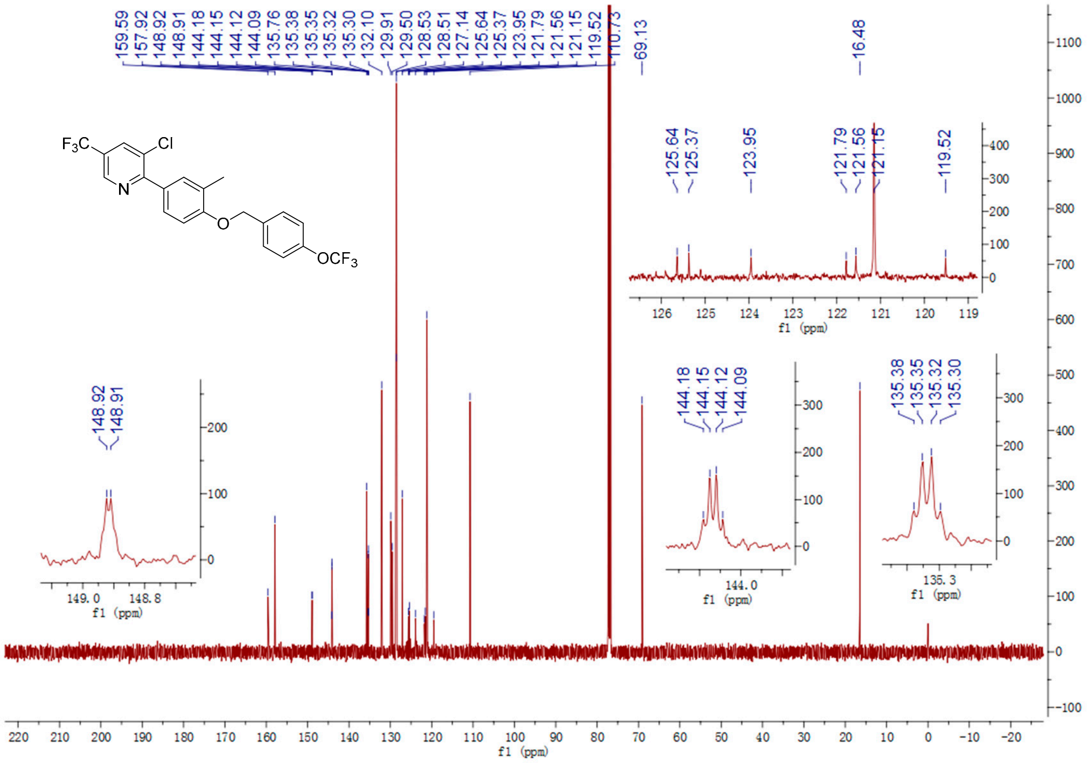

Figure S29. The ^{13}C NMR spectrum of **7g** ($CDCl_3$)

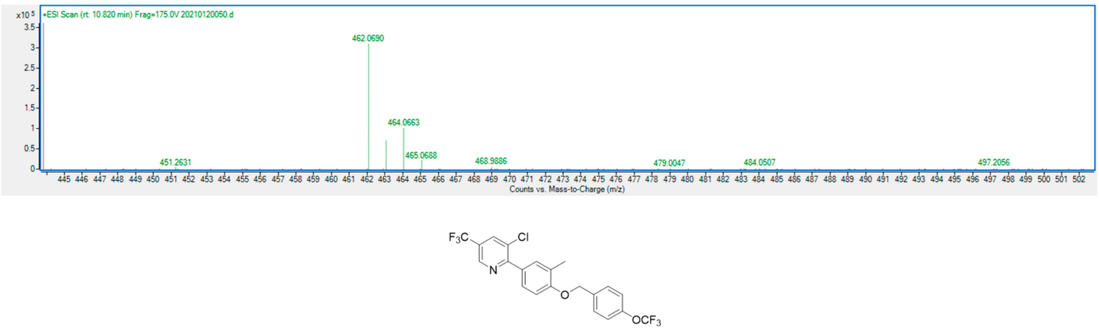

Figure S30. The HRMS spectrum of **7g**

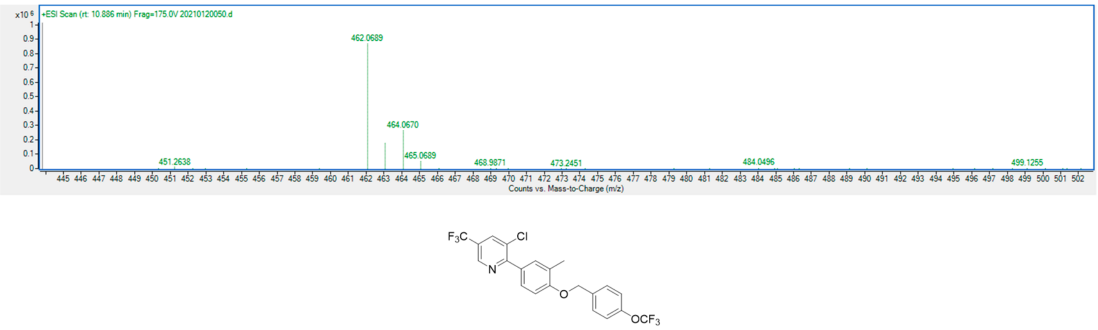

Figure S31. The HRMS spectrum of **7g**

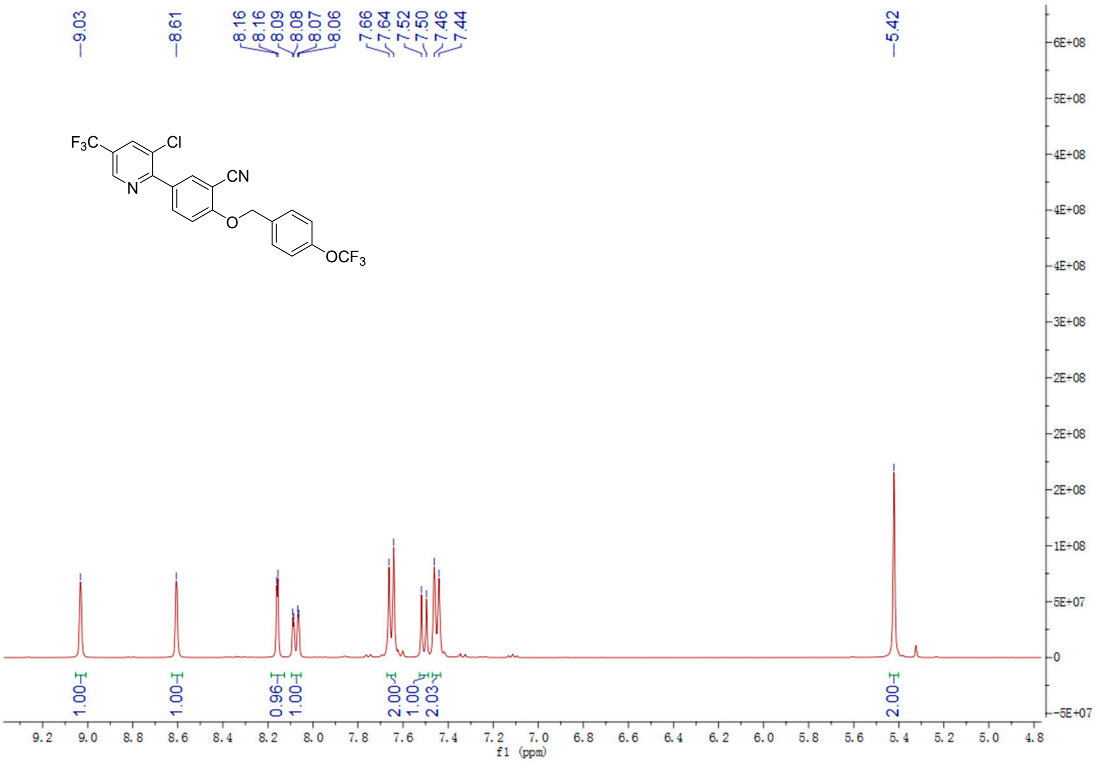

Figure S32. The ^{1}H NMR spectrum of **7h** (DMSO-d_6)

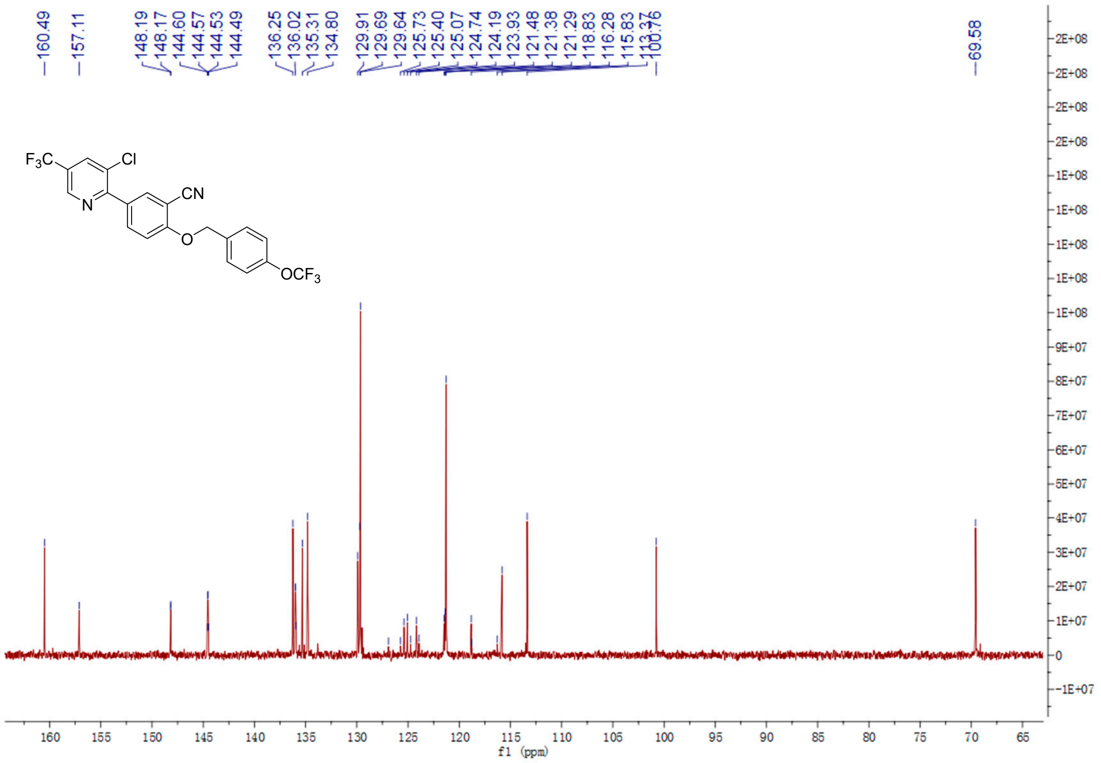

Figure S33. The ¹³C NMR spectrum of **7h** (DMSO-d_6)

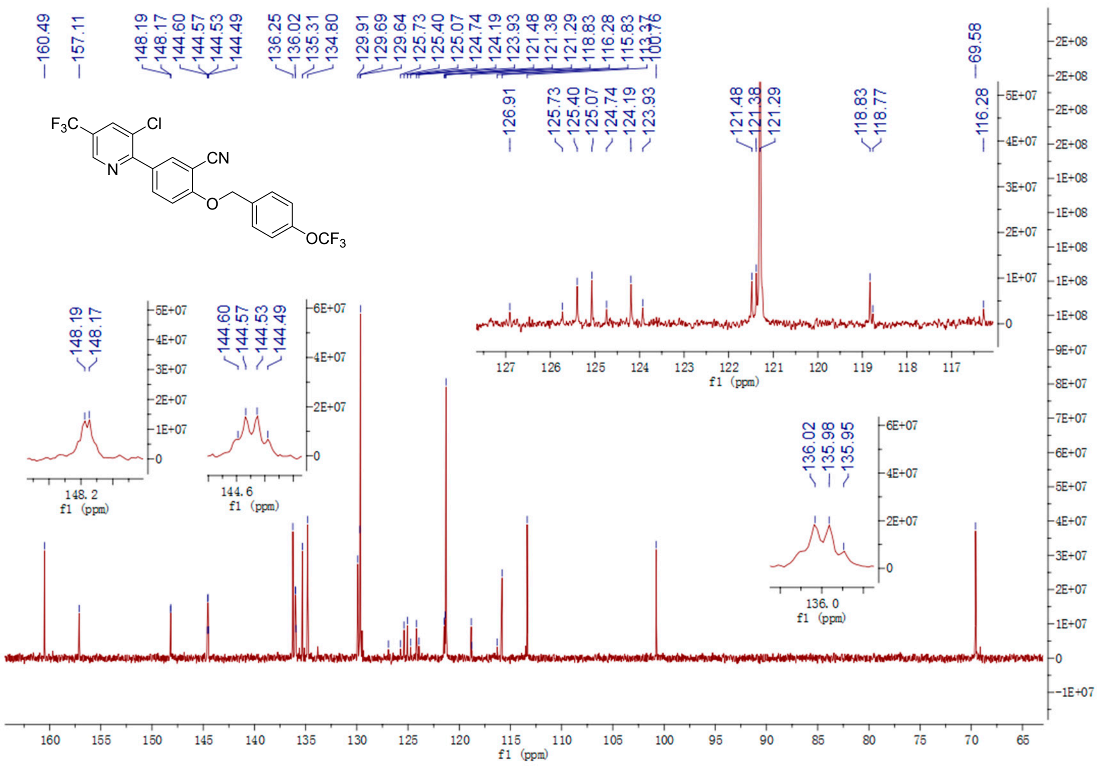

Figure S34. The ¹³C NMR spectrum of **7h** (DMSO-d_6)

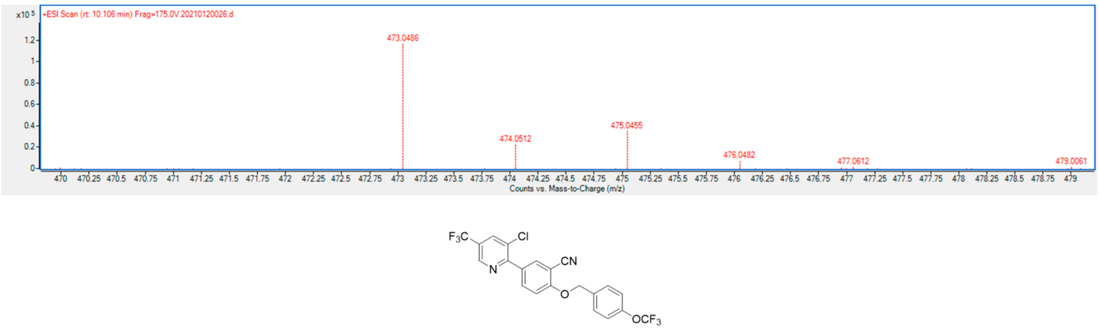

Figure S35. The HRMS spectrum of **7h**

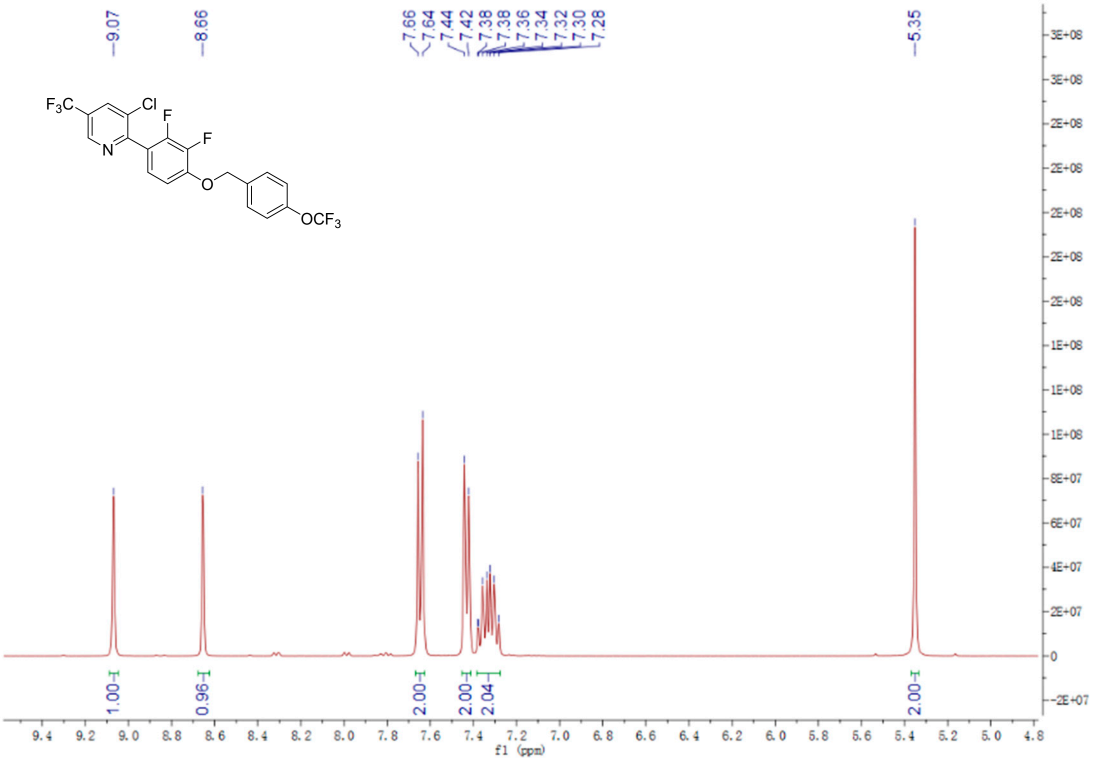

Figure S36. The ^{1}H NMR spectrum of **7i** (DMSO-d_6)

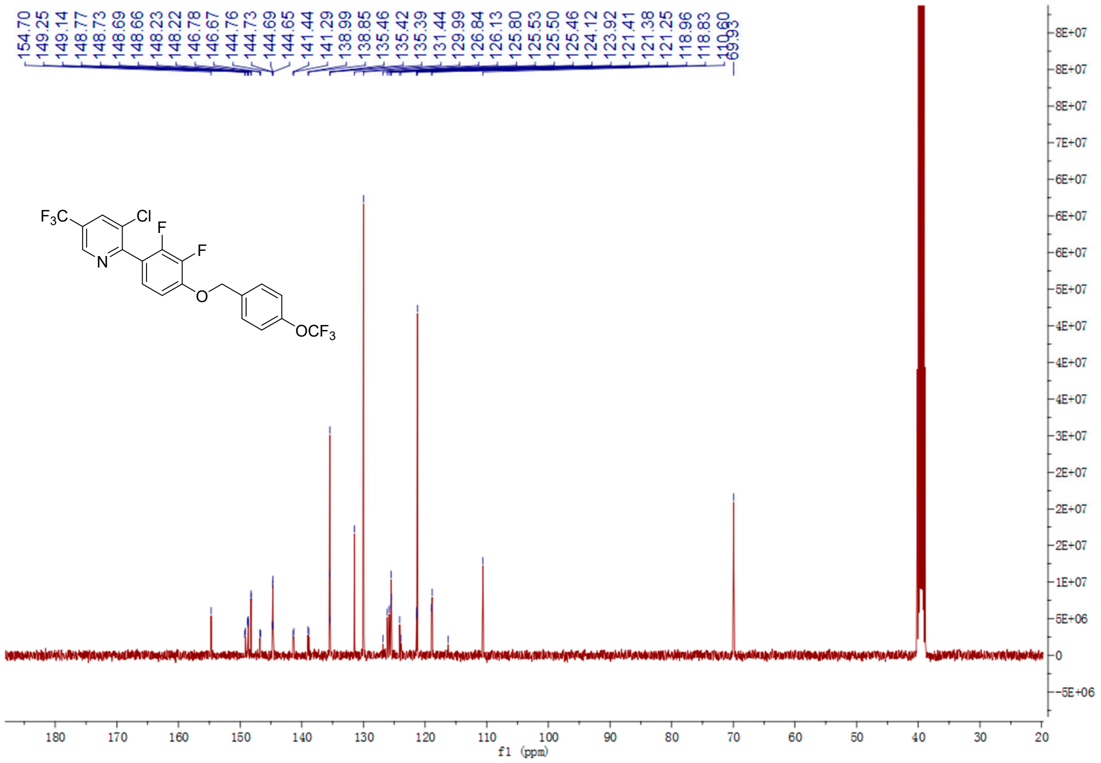

Figure S37. The ^{13}C NMR spectrum of **7i** (DMSO-d_6)

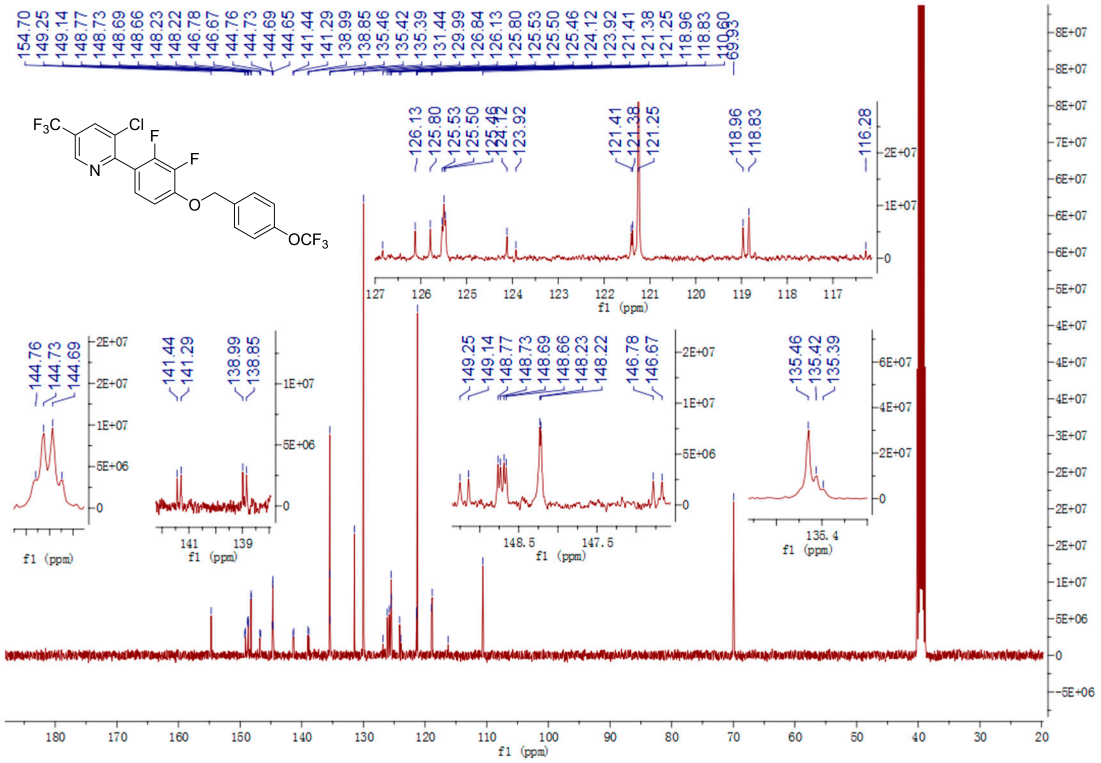

Figure S38. The ^{13}C NMR spectrum of **7i** (DMSO-d_6)

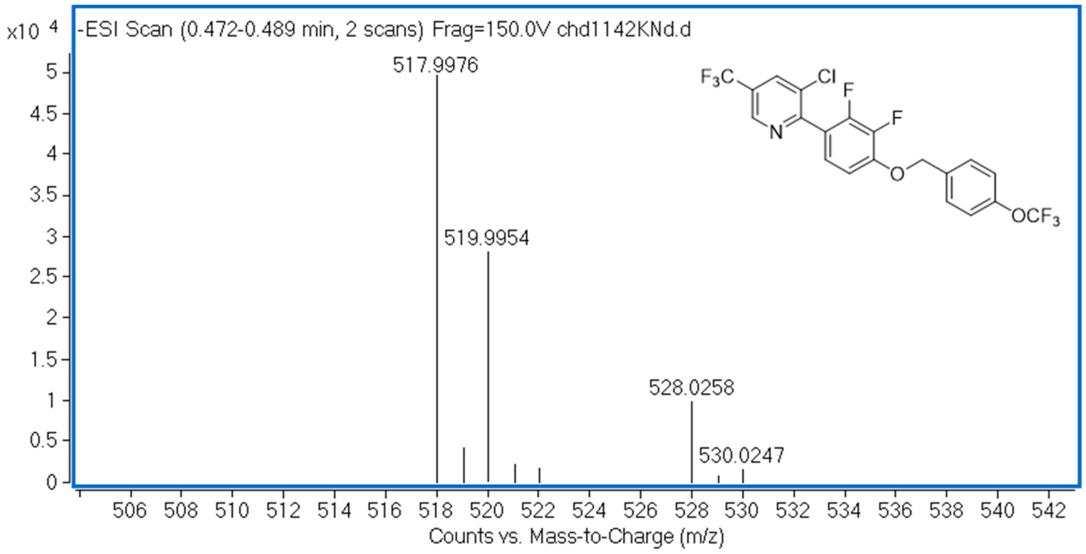

Figure S39. The HRMS spectrum of **7i**

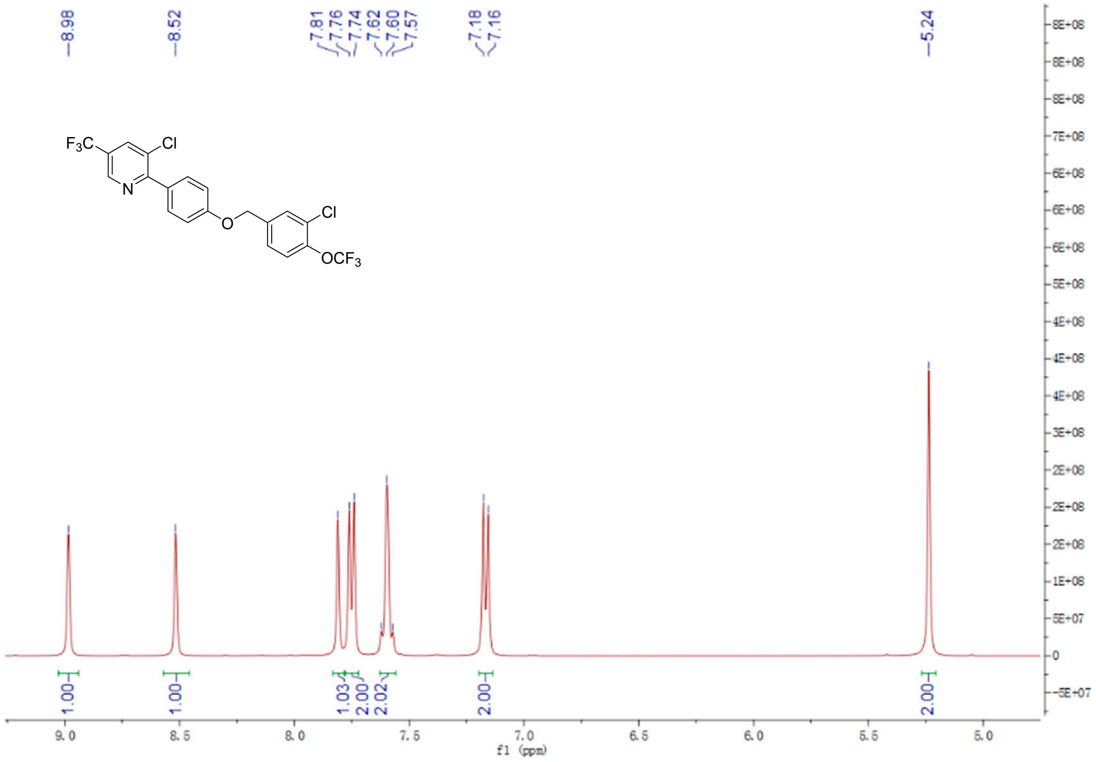

Figure S40. The ^{1}H NMR spectrum of **7j** (DMSO-d_6)

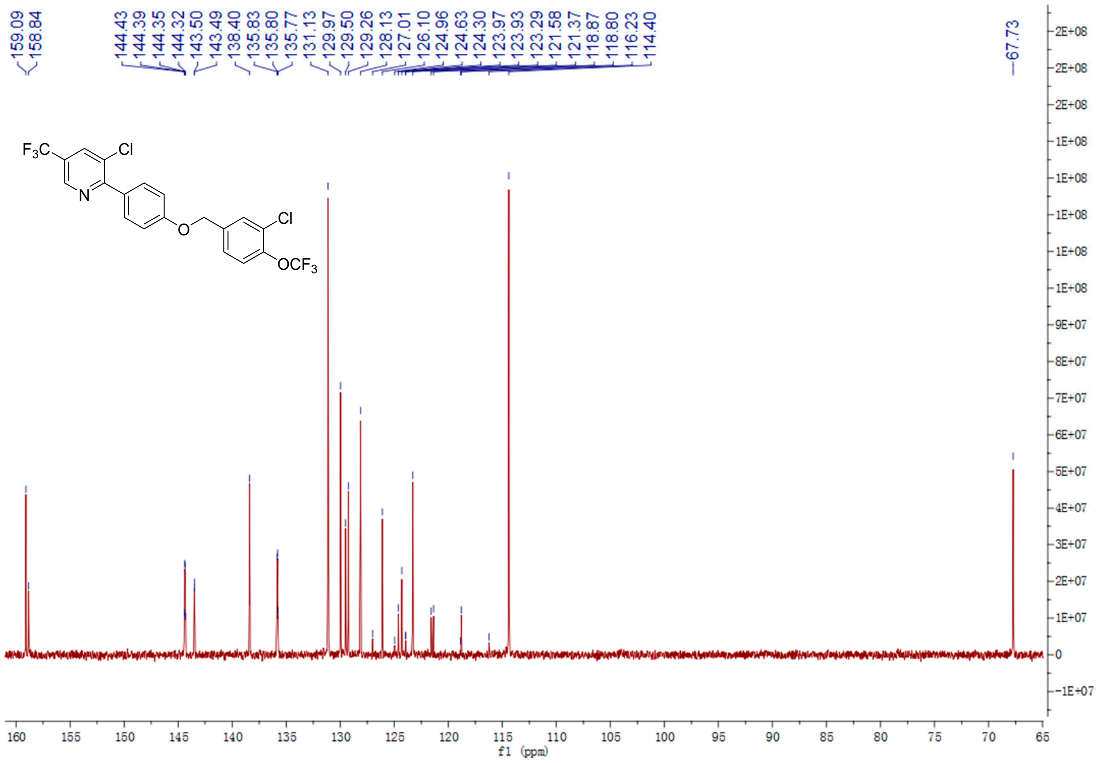

Figure S41. The ^{13}C NMR spectrum of **7j** (DMSO-d_6)

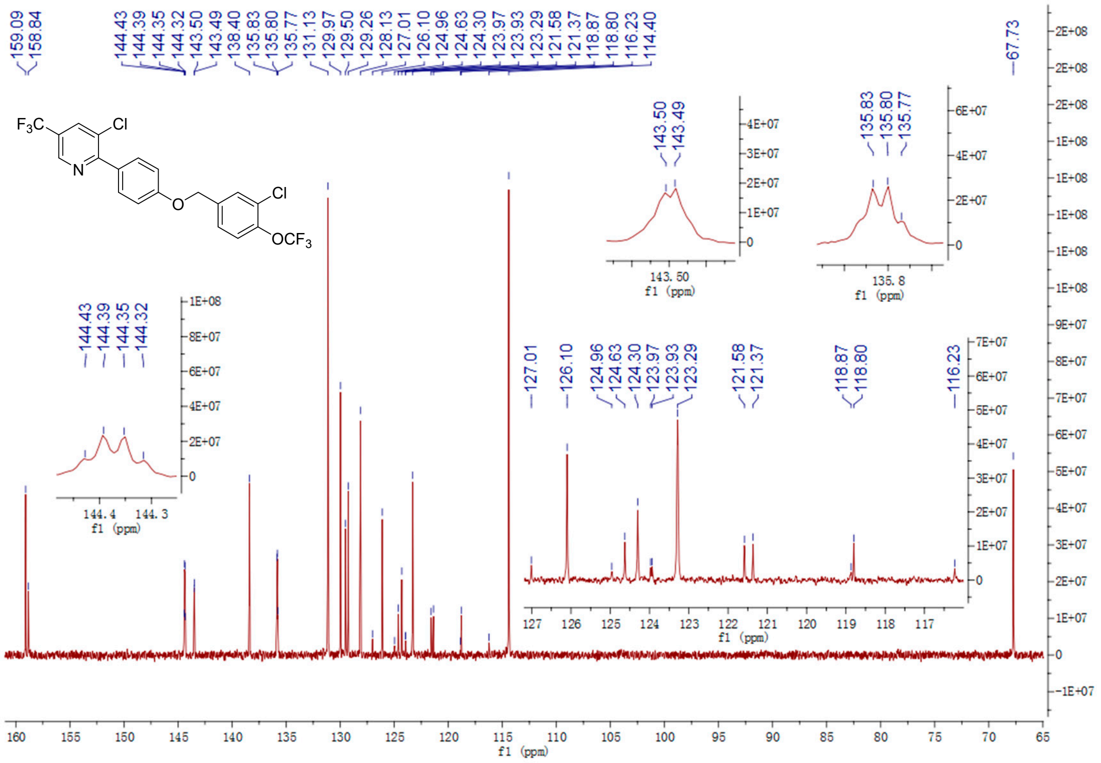

Figure S42. The ^{13}C NMR spectrum of **7j** (DMSO-d_6)

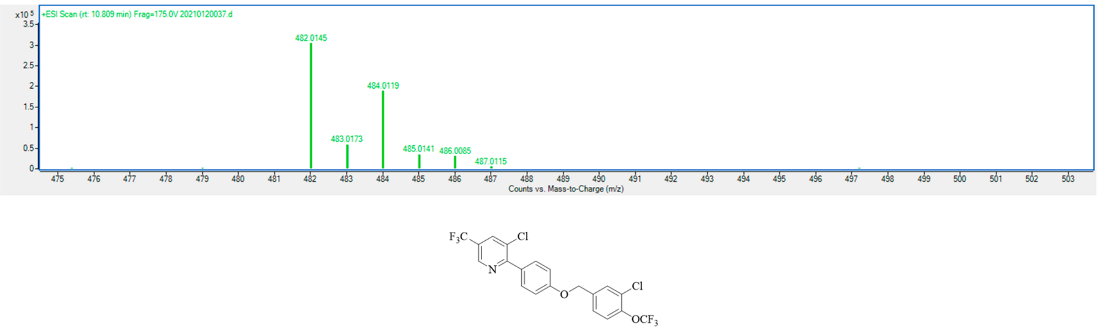

Figure S43. The HRMS spectrum of **7j**

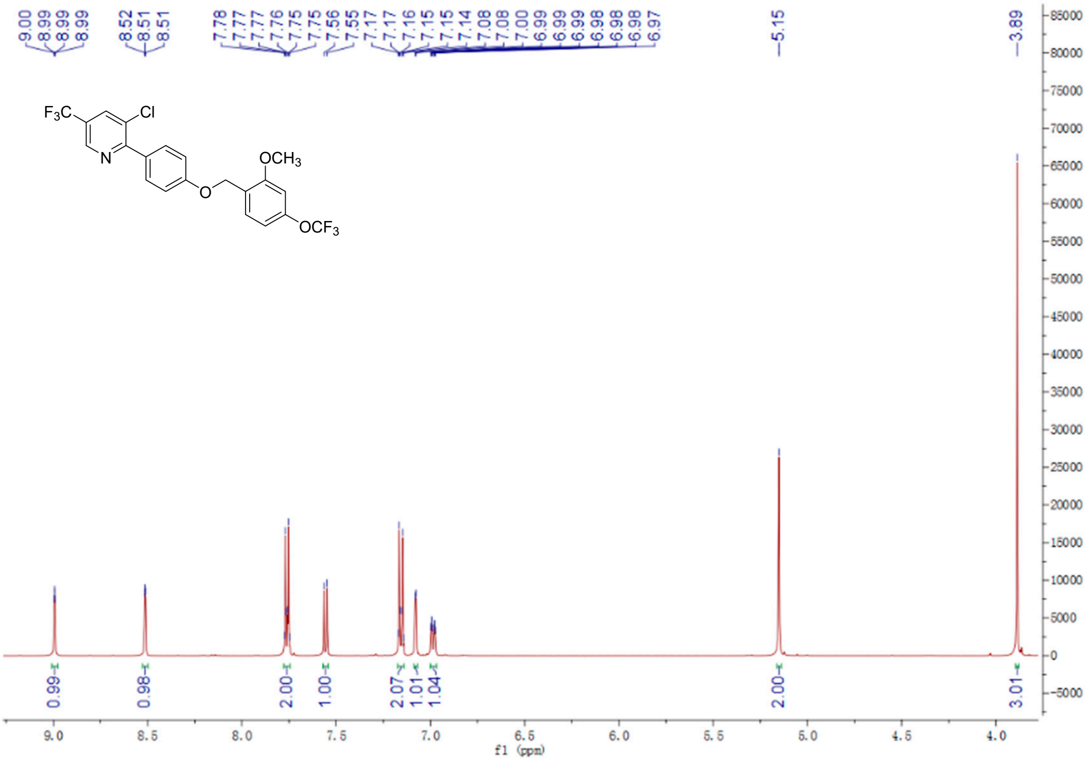

Figure S44. The ^{1}H NMR spectrum of **7k** (DMSO-d_6)

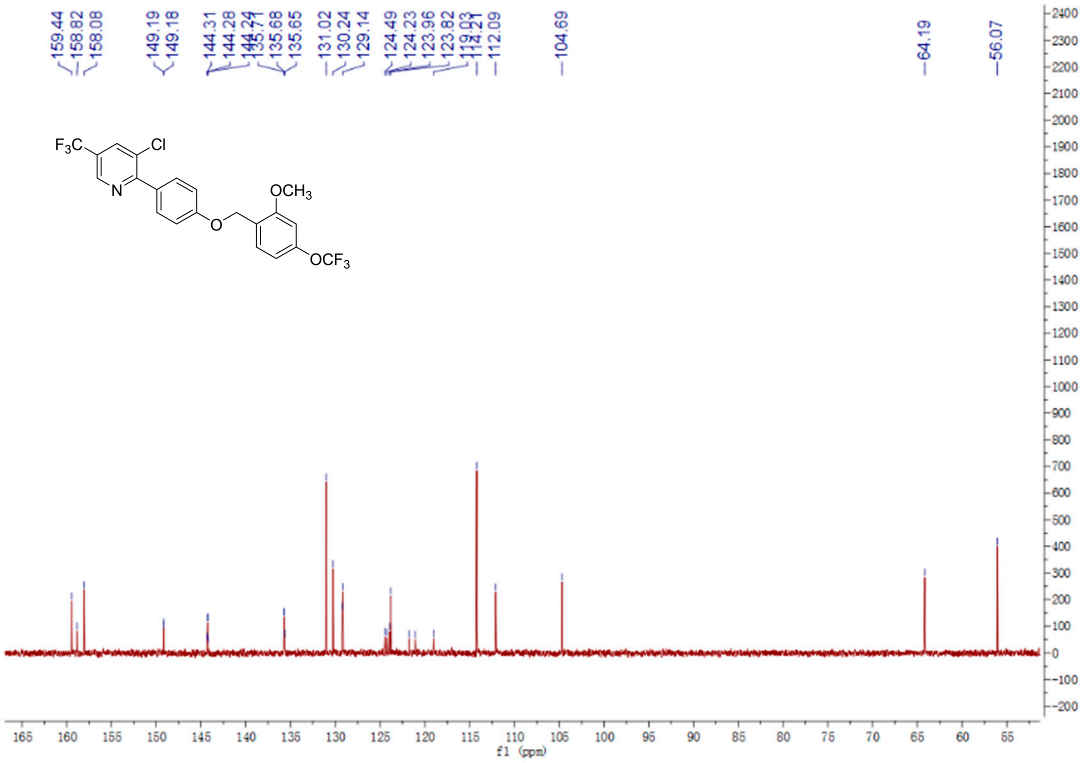

Figure S45. The ^{13}C NMR spectrum of **7k** (DMSO-*d*$_6$)

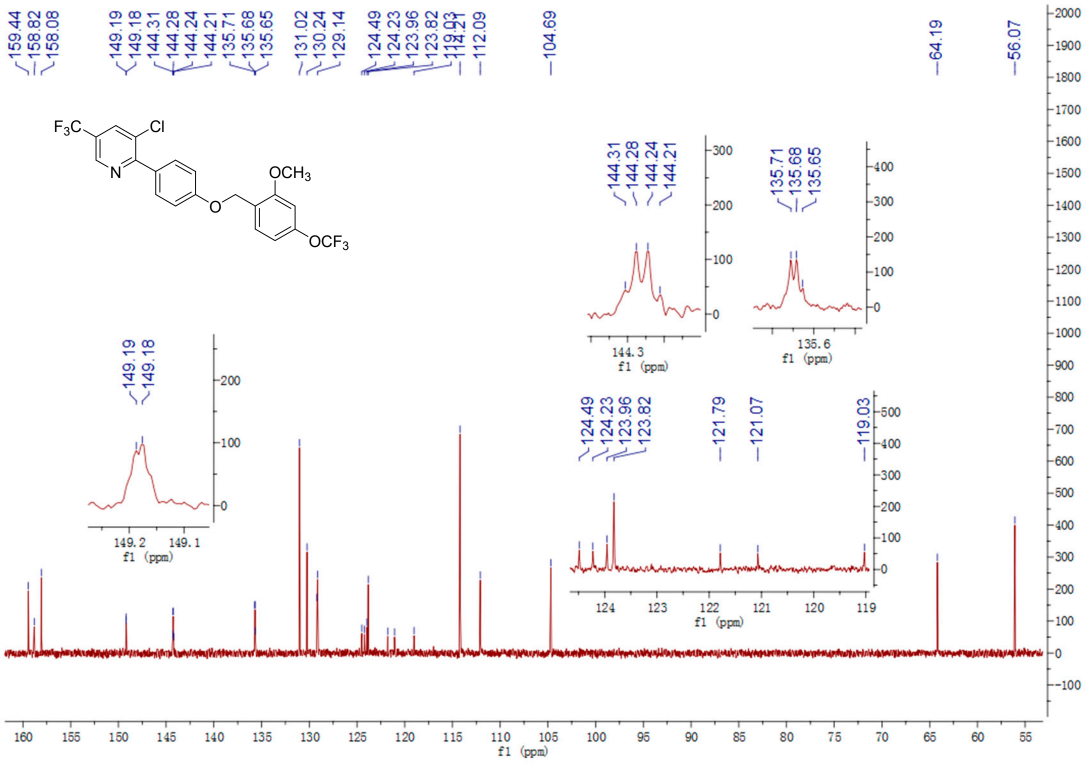

Figure S46. The ^{13}C NMR spectrum of **7k** (DMSO-*d*$_6$)

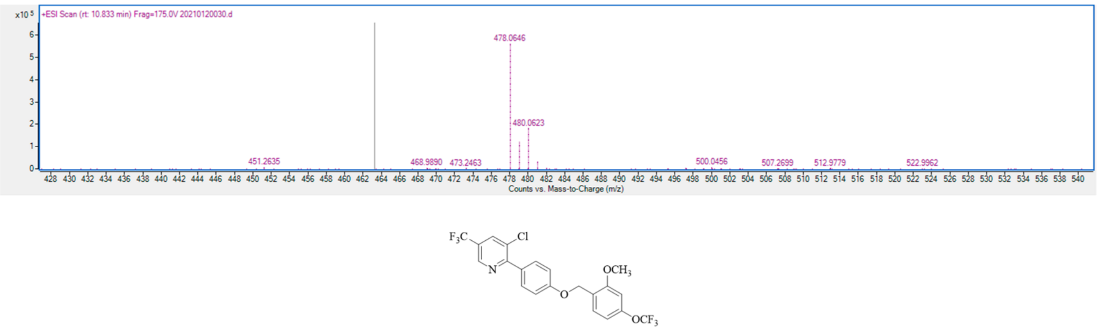

Figure S47. The HRMS spectrum of **7k**

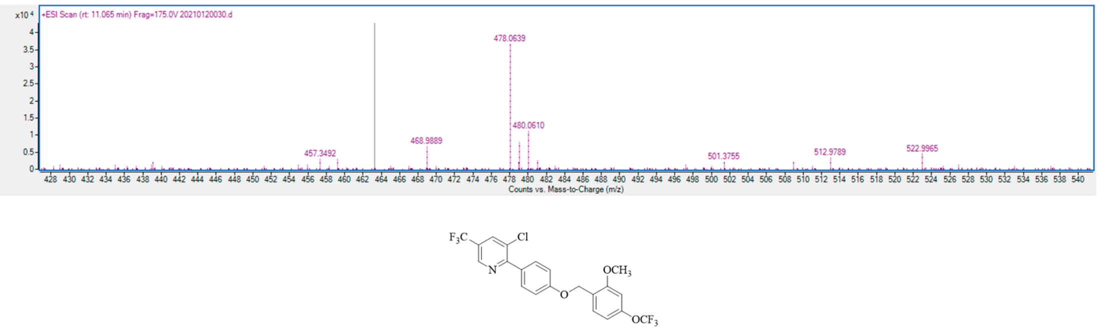

Figure S48. The HRMS spectrum of **7k**

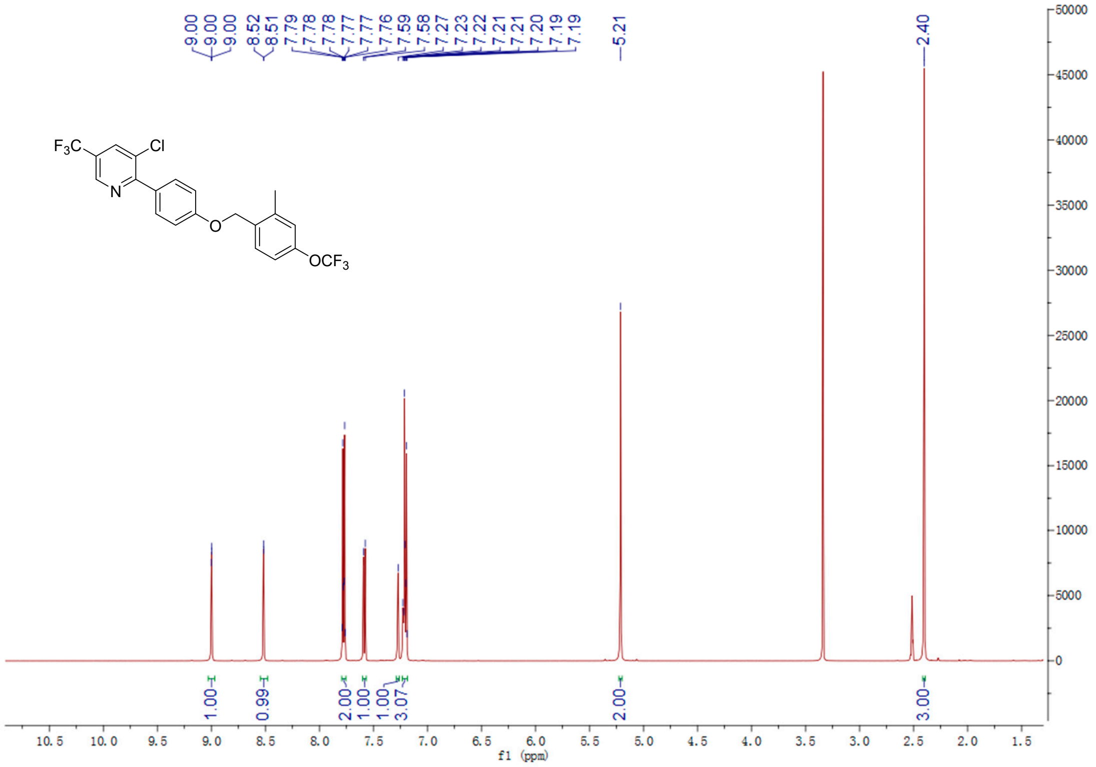

Figure S49. The ^{1}H NMR spectrum of **7l** (DMSO-d_6)

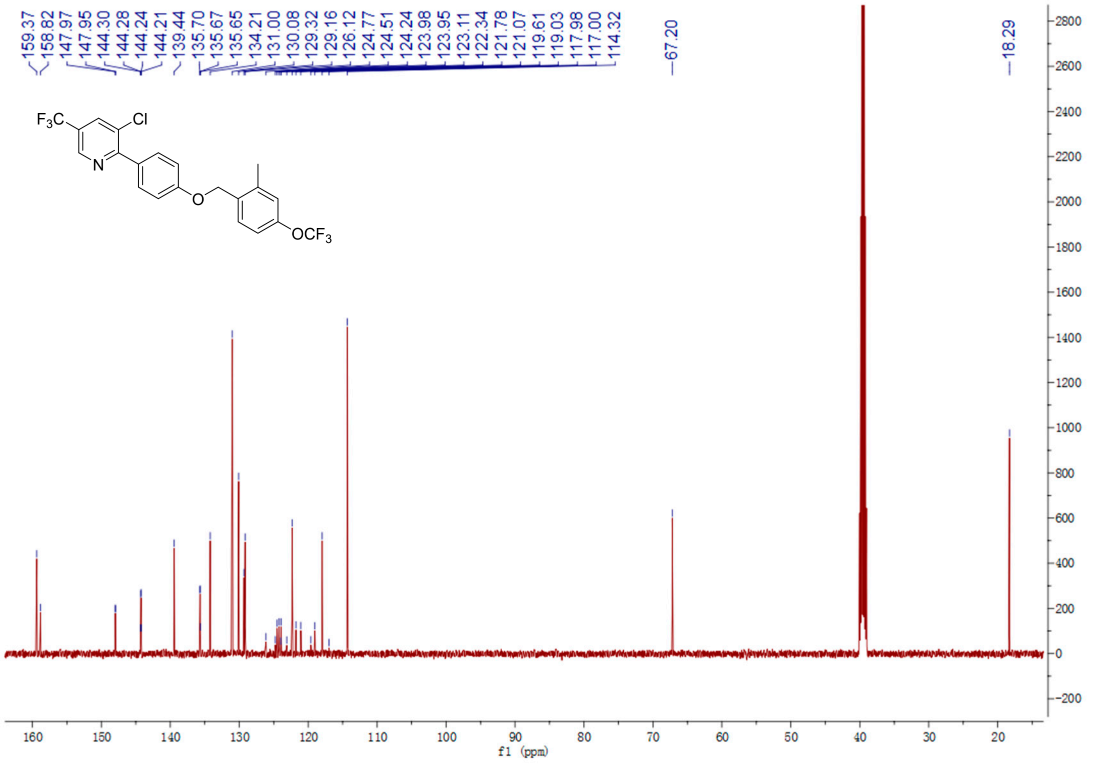

Figure S50. The ^{13}C NMR spectrum of **7l** (DMSO-d_6)

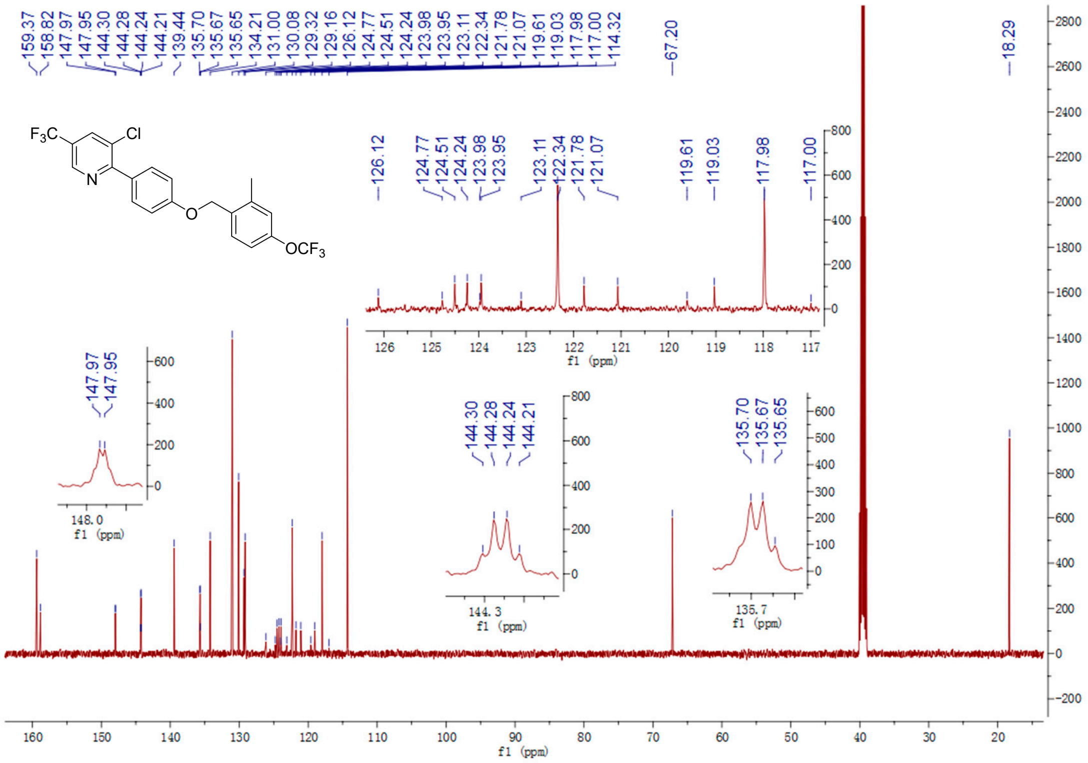

Figure S51. The ^{13}C NMR spectrum of **7l** (DMSO-d_6)

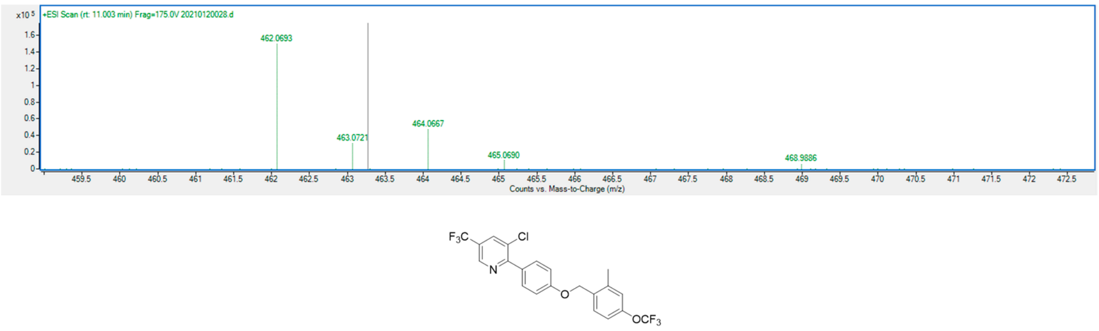

Figure S52. The HRMS spectrum of **7l**

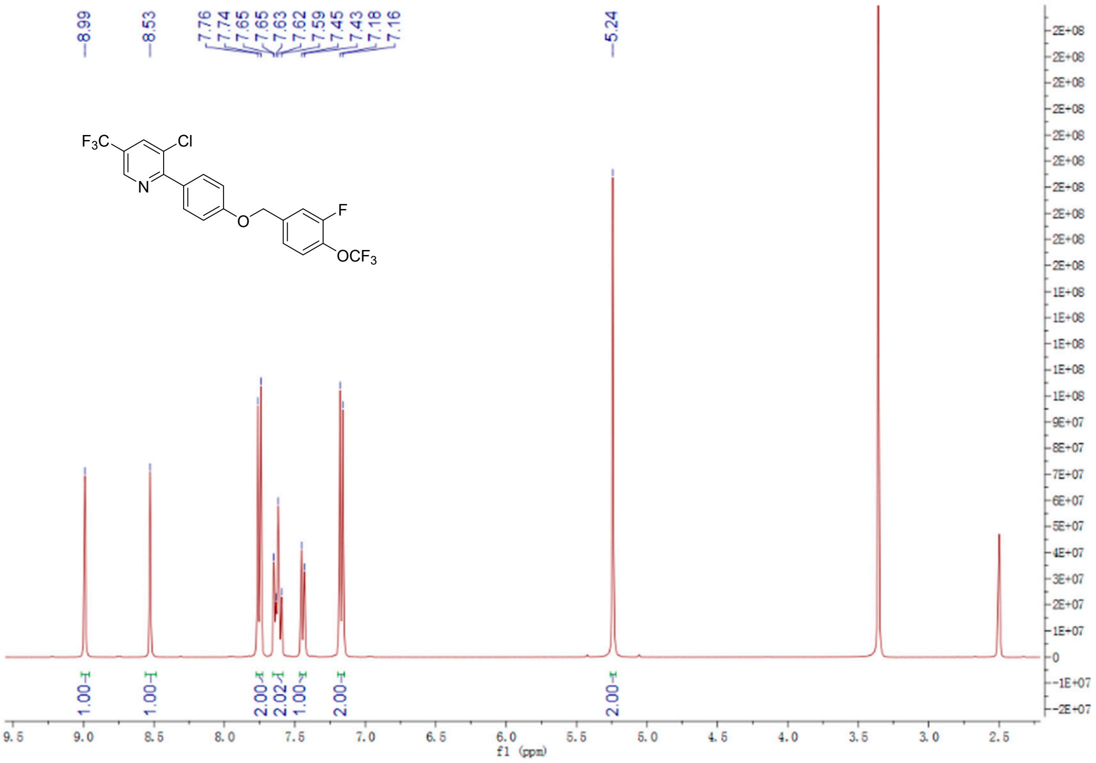

Figure S53. The ^{1}H NMR spectrum of **7m** (DMSO-d_6)

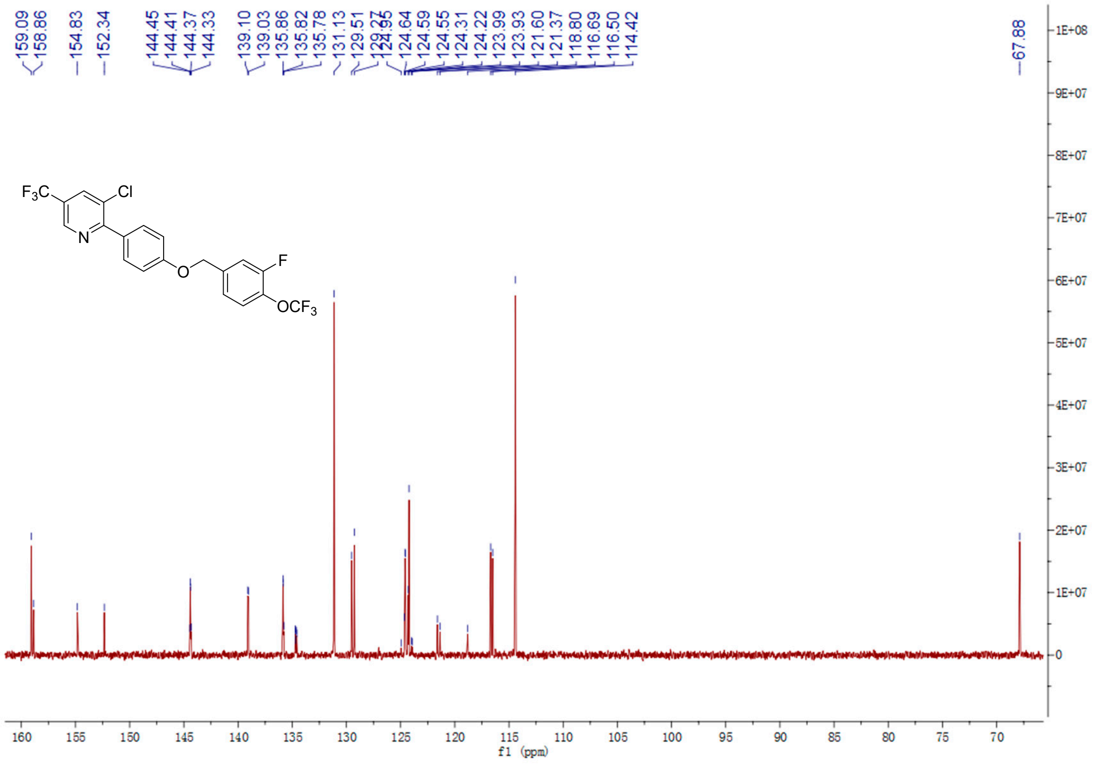

Figure S54. The ^{13}C NMR spectrum of **7m** (DMSO-d_6)

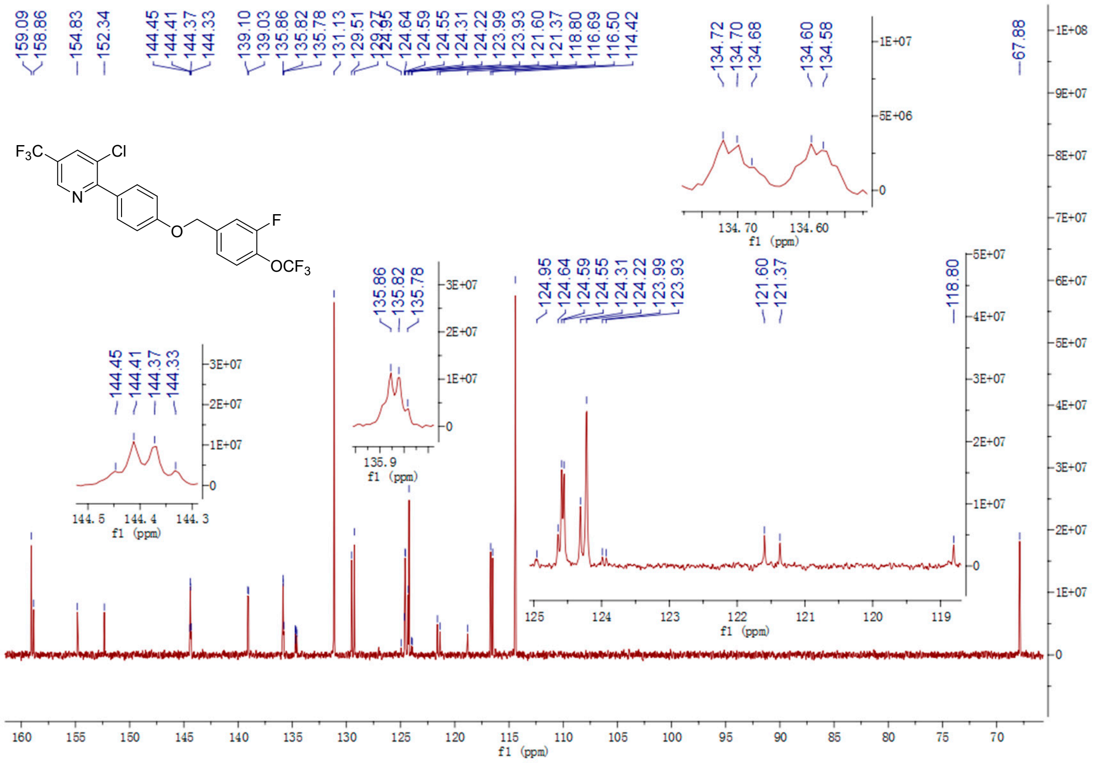

Figure S55. The ^{13}C NMR spectrum of **7m** (DMSO-d_6)

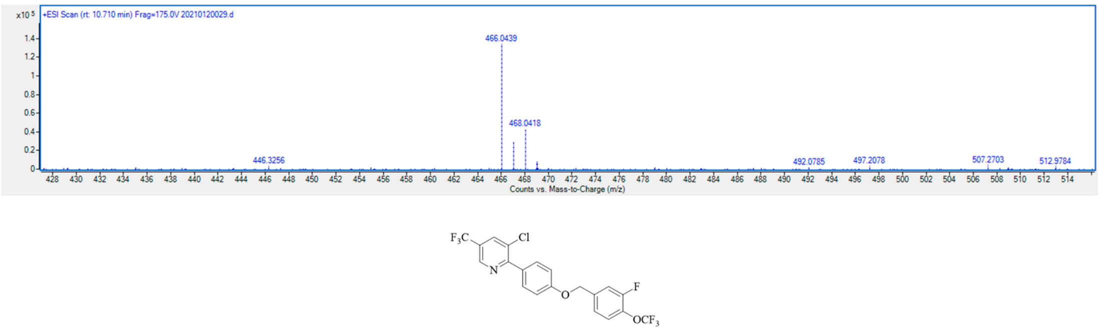

Figure S56. The HRMS spectrum of **7m**

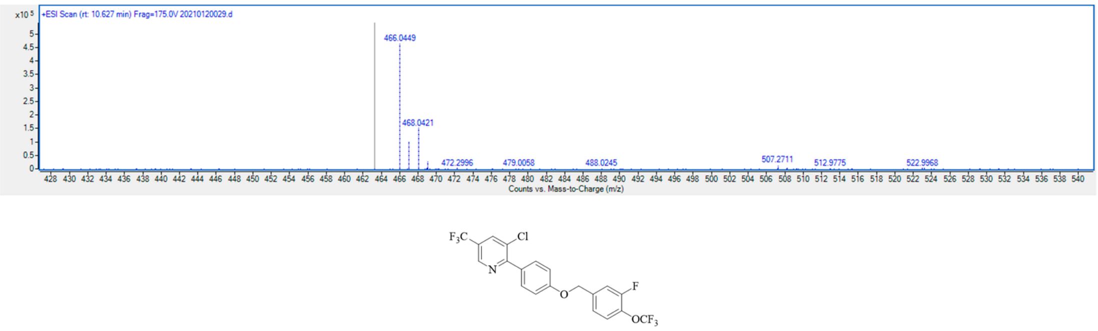

Figure S57. The HRMS spectrum of **7m**

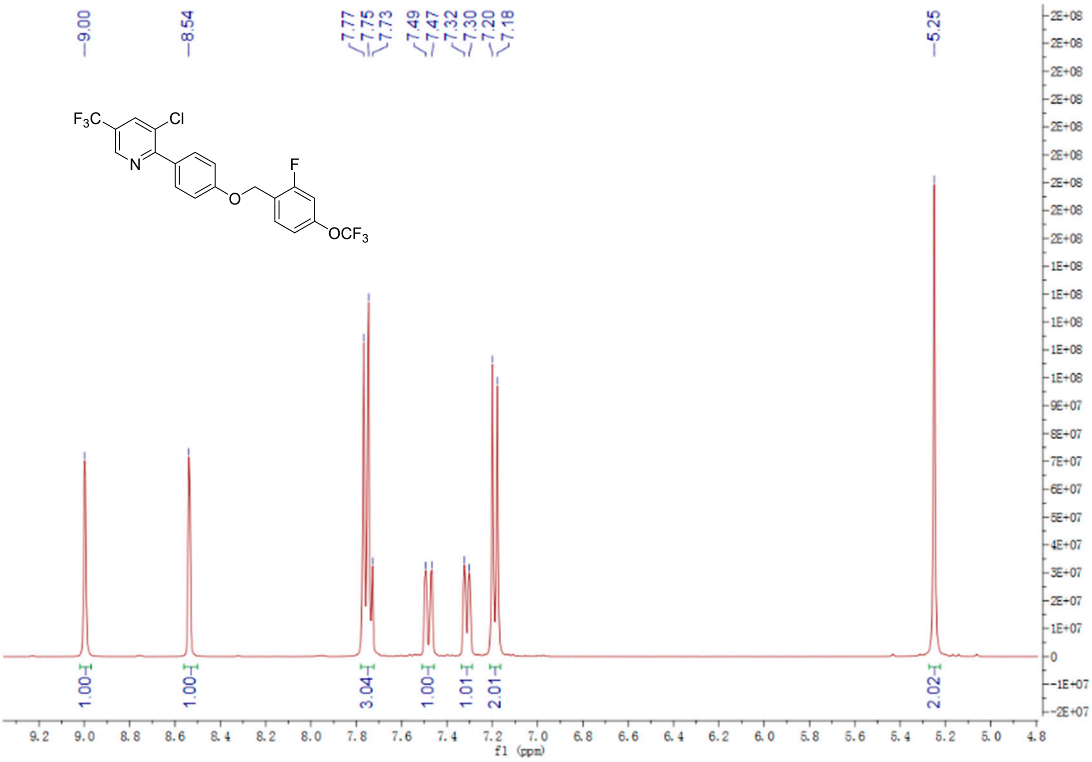

Figure S58. The ^{1}H NMR spectrum of **7n** (DMSO-d_6)

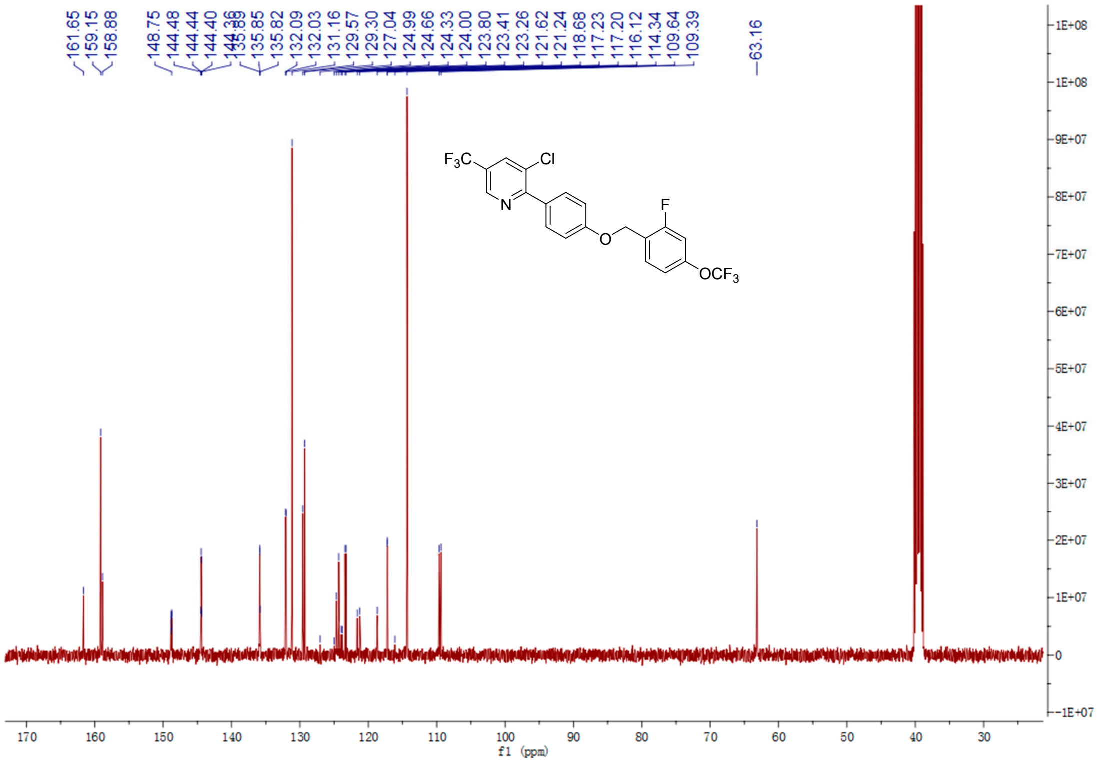

Figure S59. The ^{13}C NMR spectrum of **7n** (DMSO-*d*$_6$)

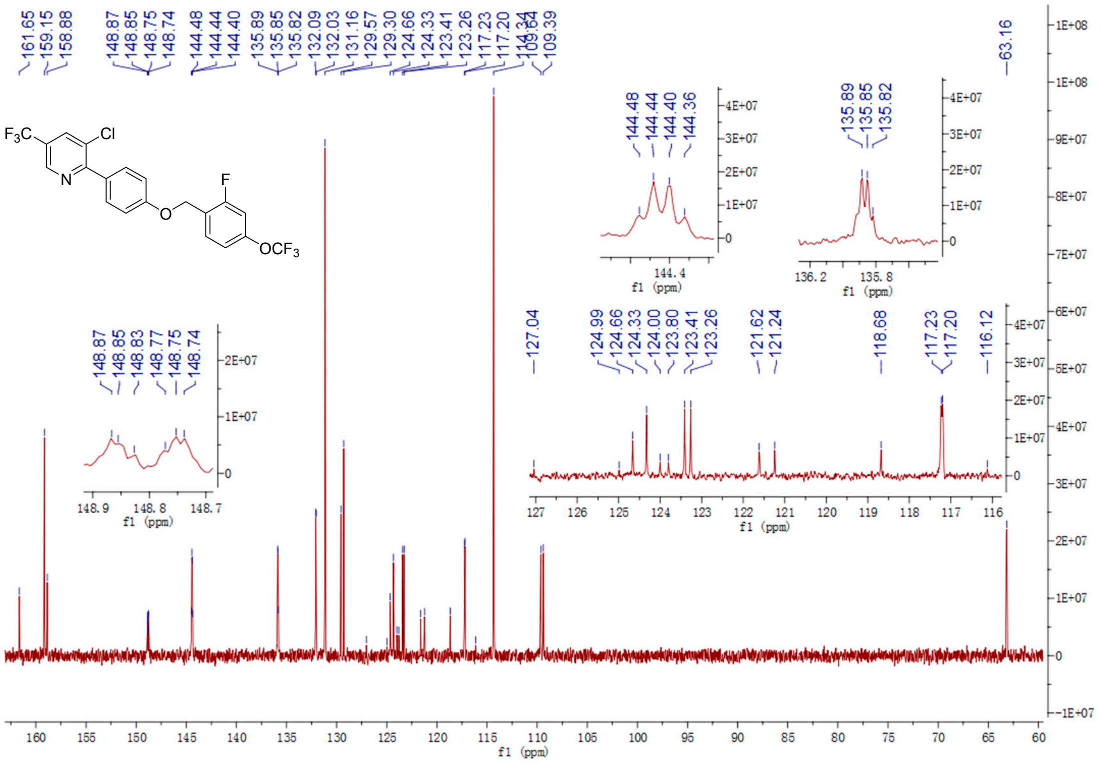

Figure S60. The ^{13}C NMR spectrum of **7n** (DMSO-*d*$_6$)

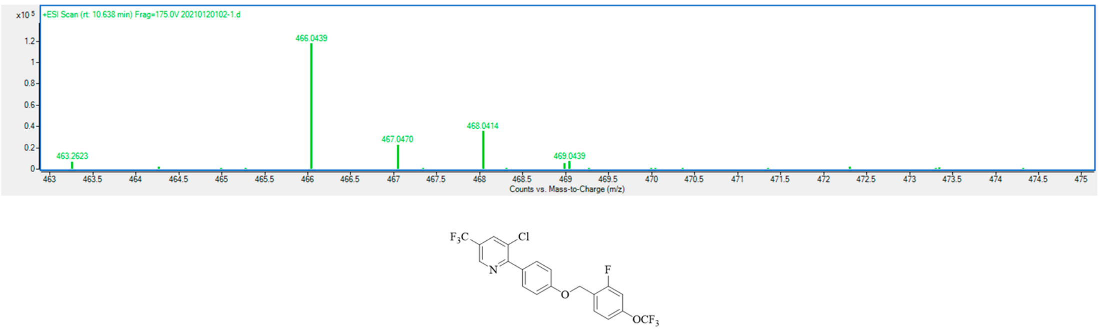

Figure S61. The HRMS spectrum of **7n**

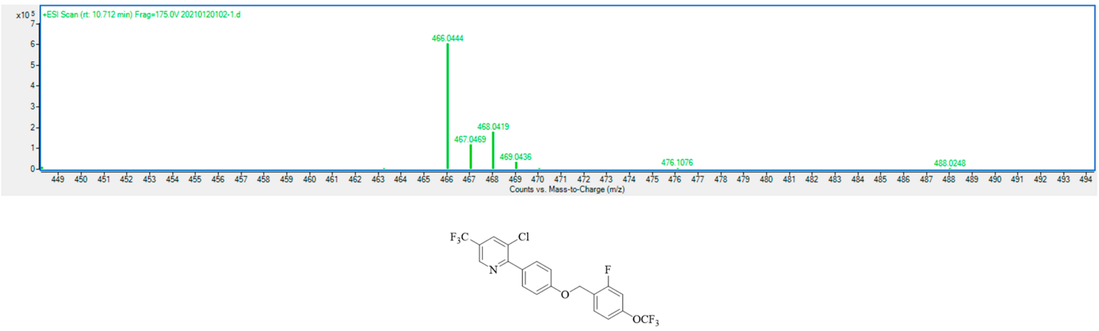

Figure S62. The HRMS spectrum of **7n**

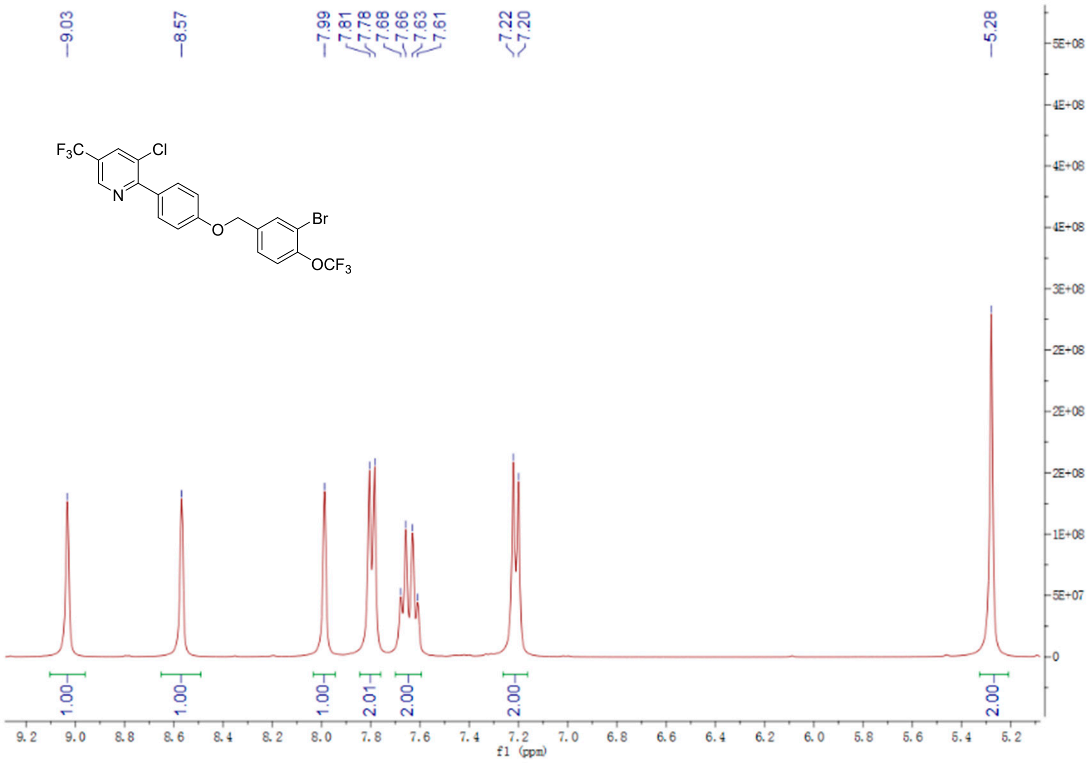

Figure S63. The ^{1}H NMR spectrum of **7o** (DMSO-d_6)

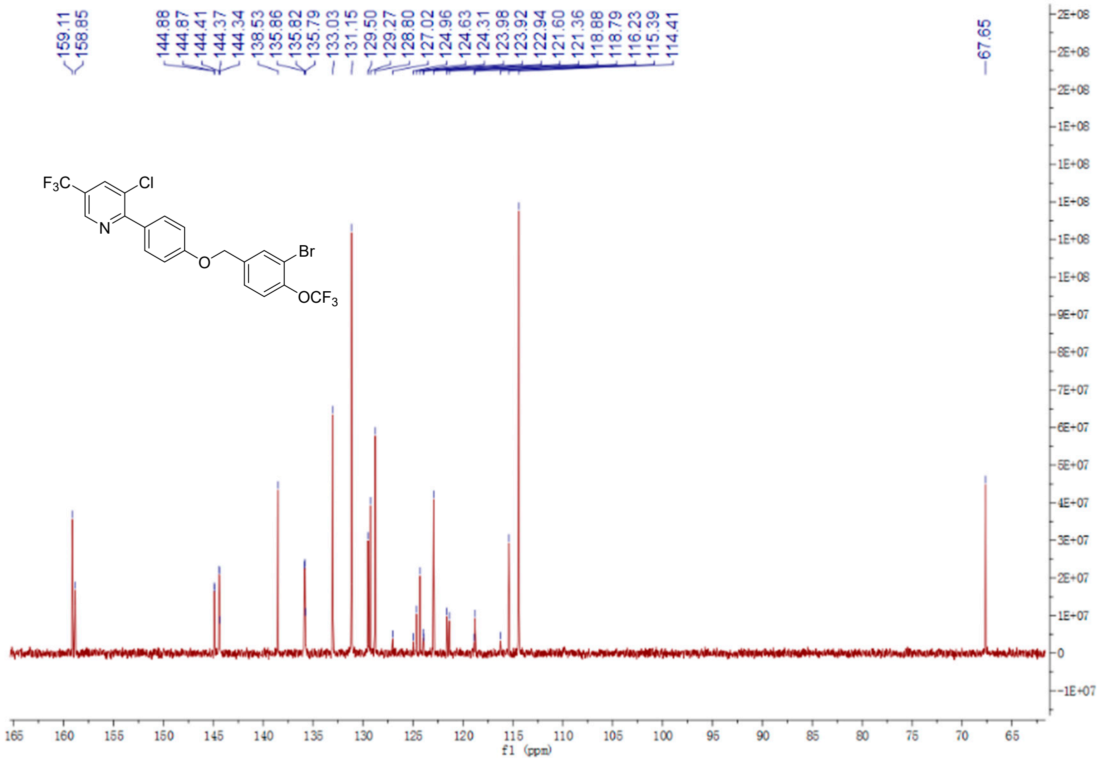

Figure S64. The ^{13}C NMR spectrum of **7o** (DMSO-d_6)

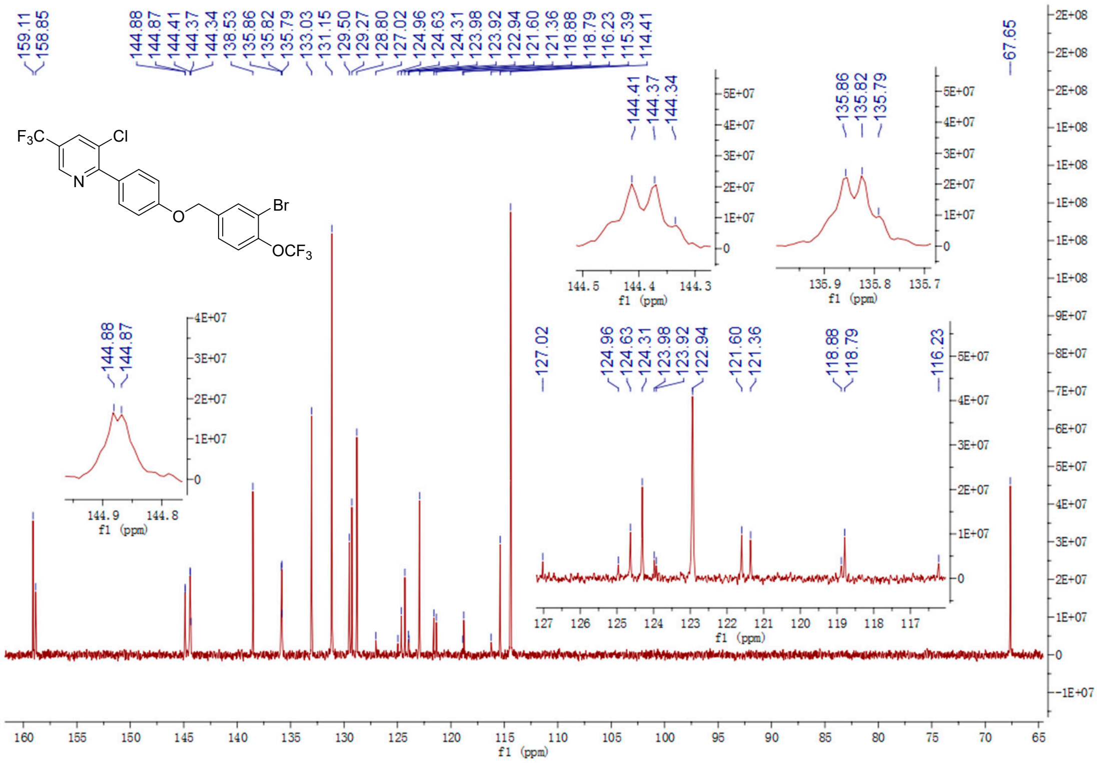

Figure S65. The ^{13}C NMR spectrum of **7o** (DMSO-d_6)

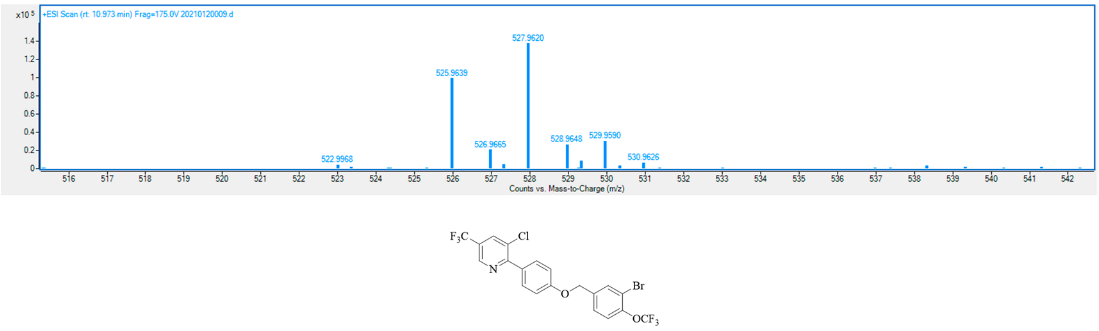

Figure S66. The HRMS spectrum of **7o**

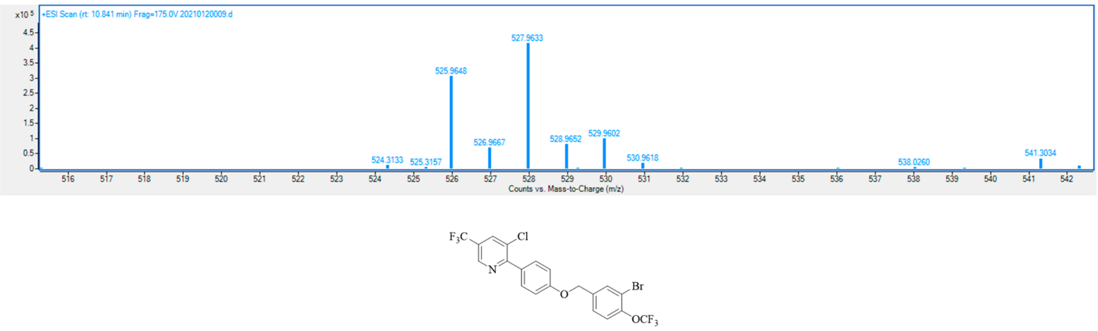

Figure S67. The HRMS spectrum of **7o**

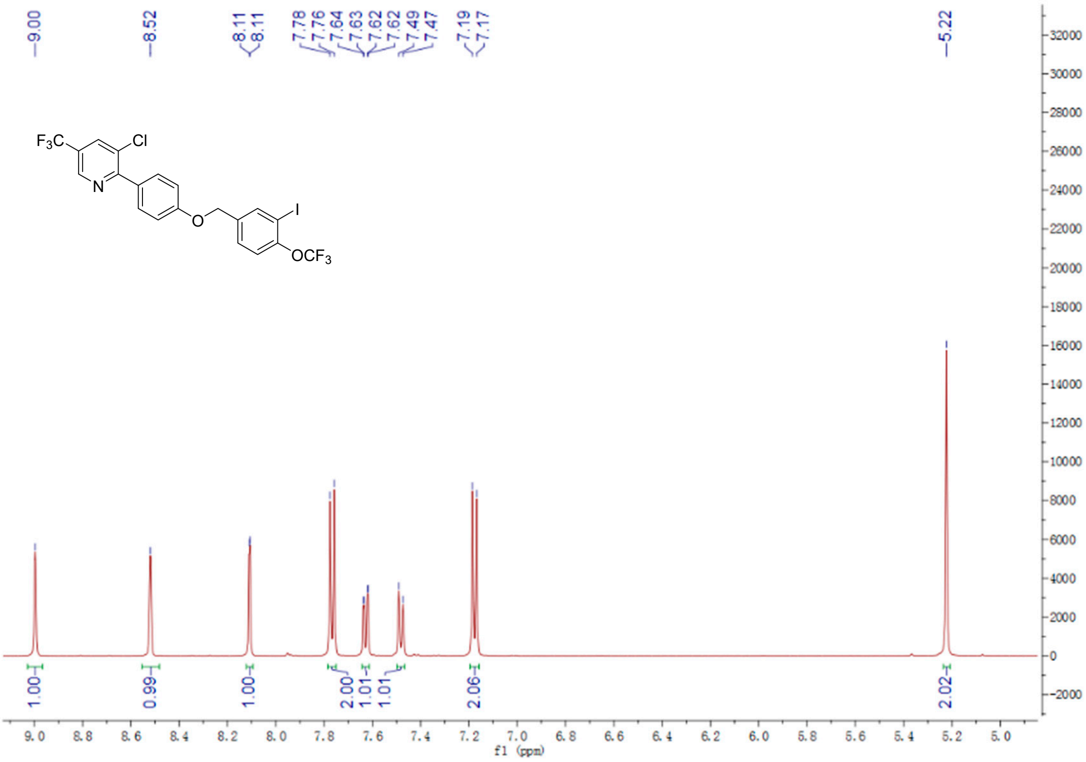

Figure S68. The ^{1}H NMR spectrum of **7p** (DMSO-d_6)

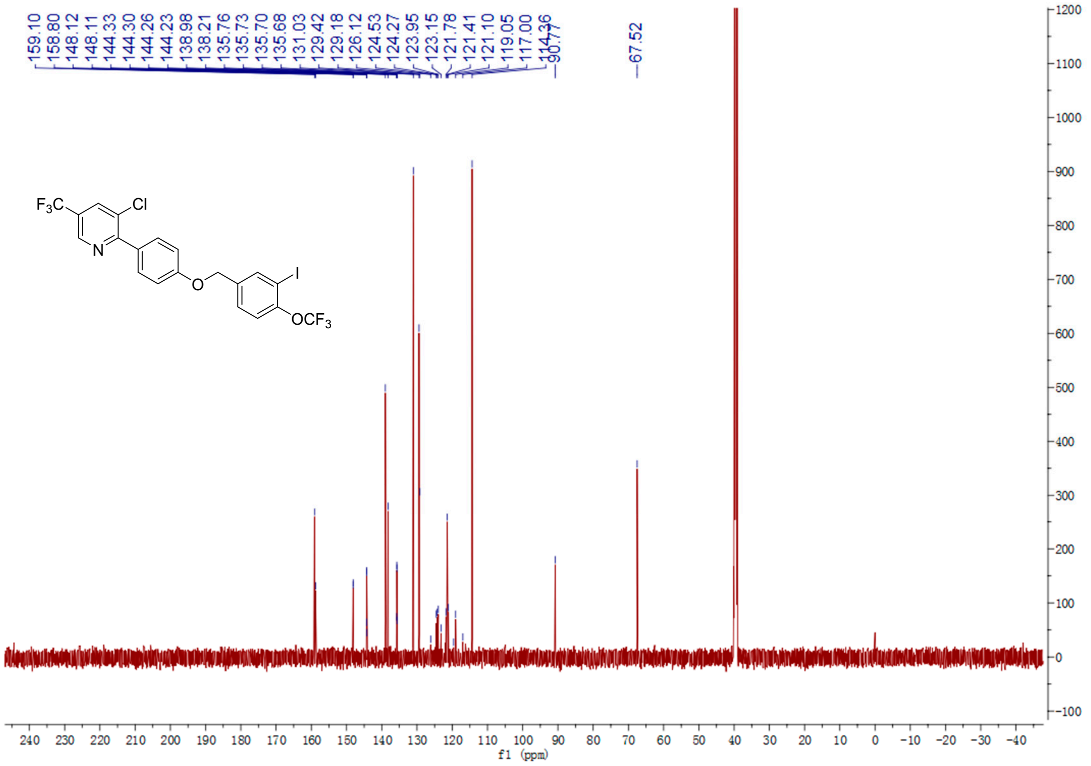

Figure S69. The ^{13}C NMR spectrum of **7p** (DMSO-d_6)

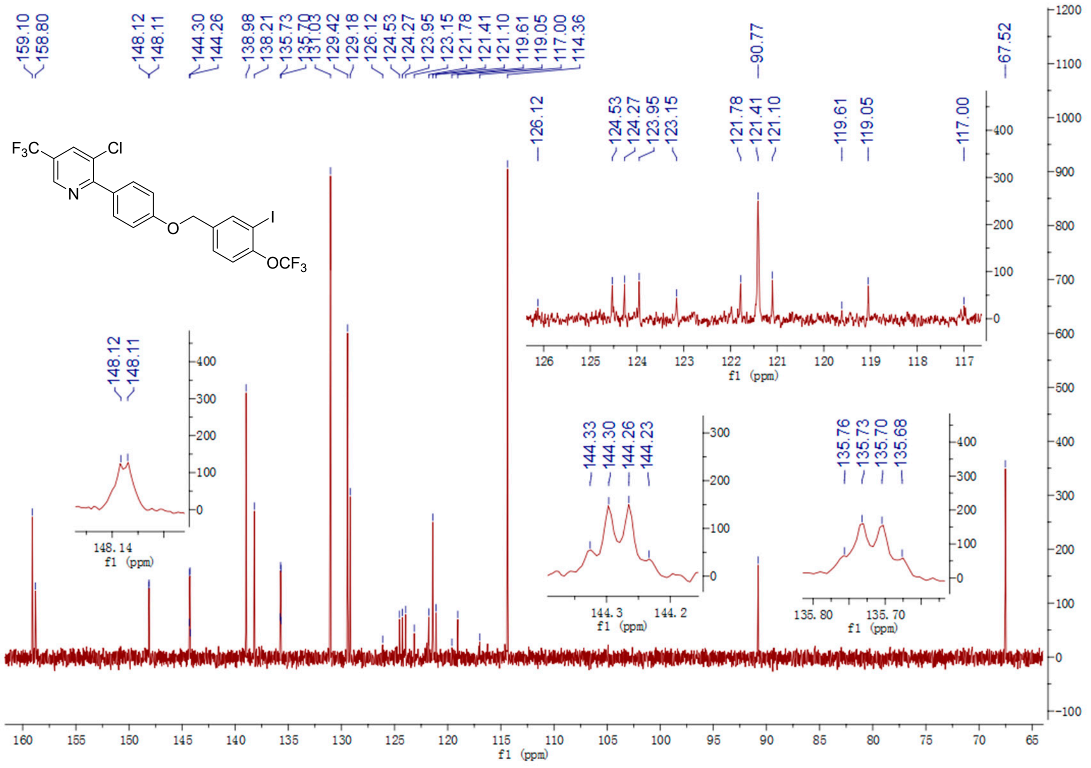

Figure S70. The ^{13}C NMR spectrum of **7p** (DMSO-d_6)

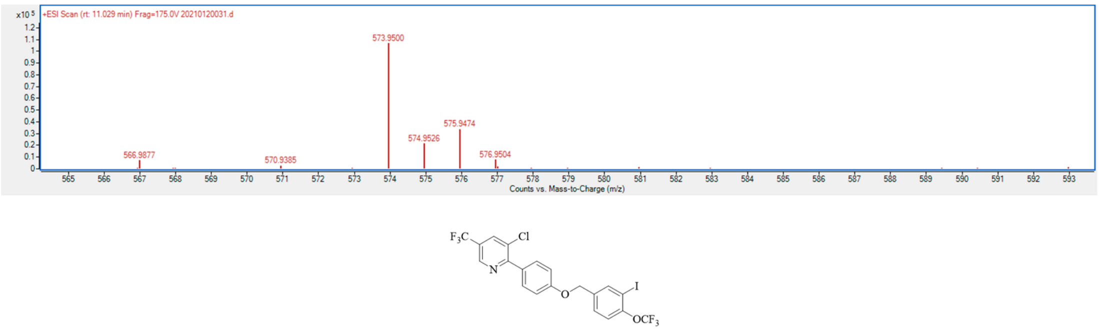

Figure S71. The HRMS spectrum of **7p**

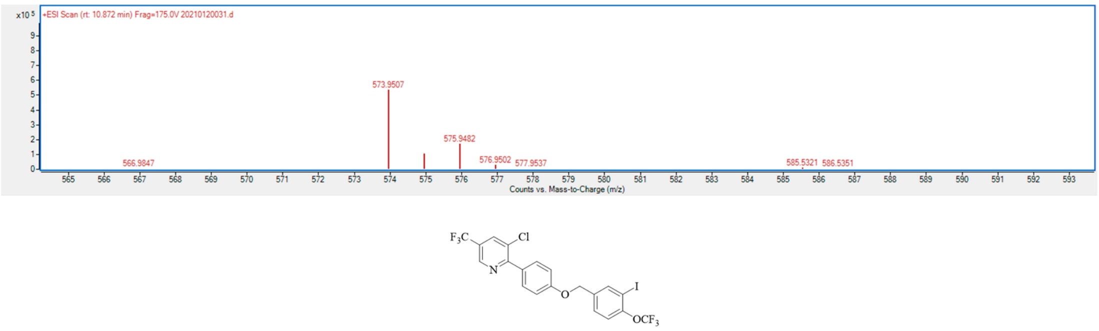

Figure S72. The HRMS spectrum of **7p**

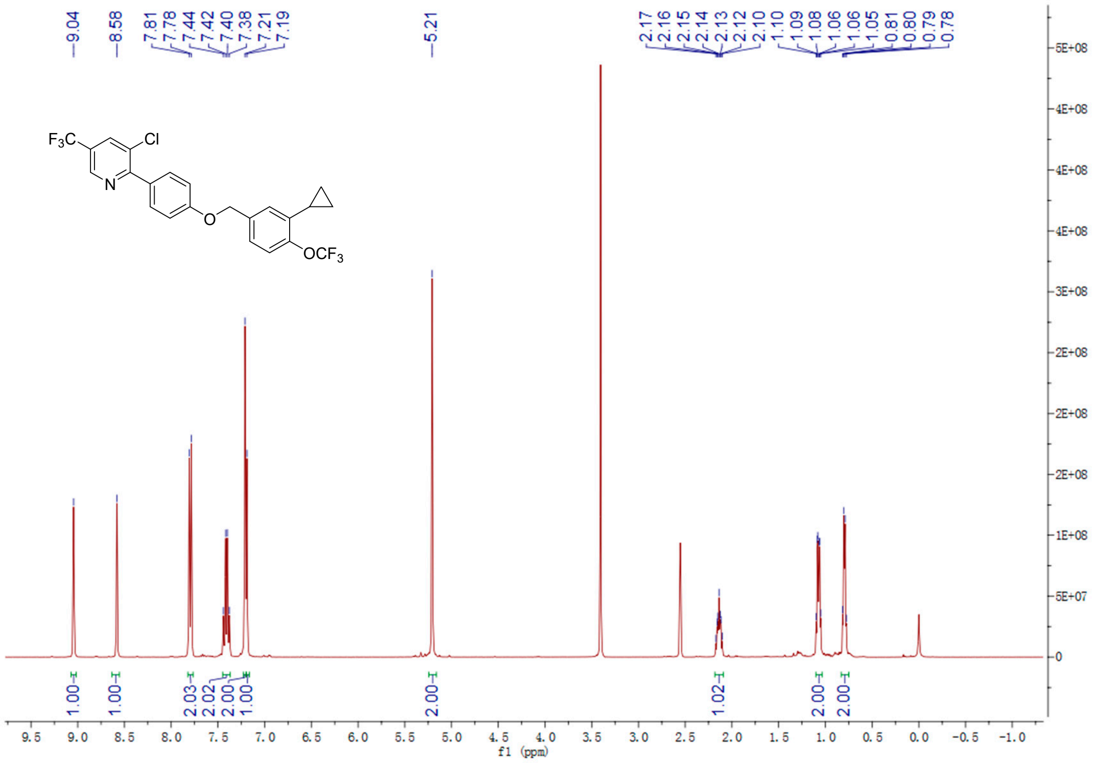

Figure S73. The ^{1}H NMR spectrum of **7q** (DMSO-d_6)

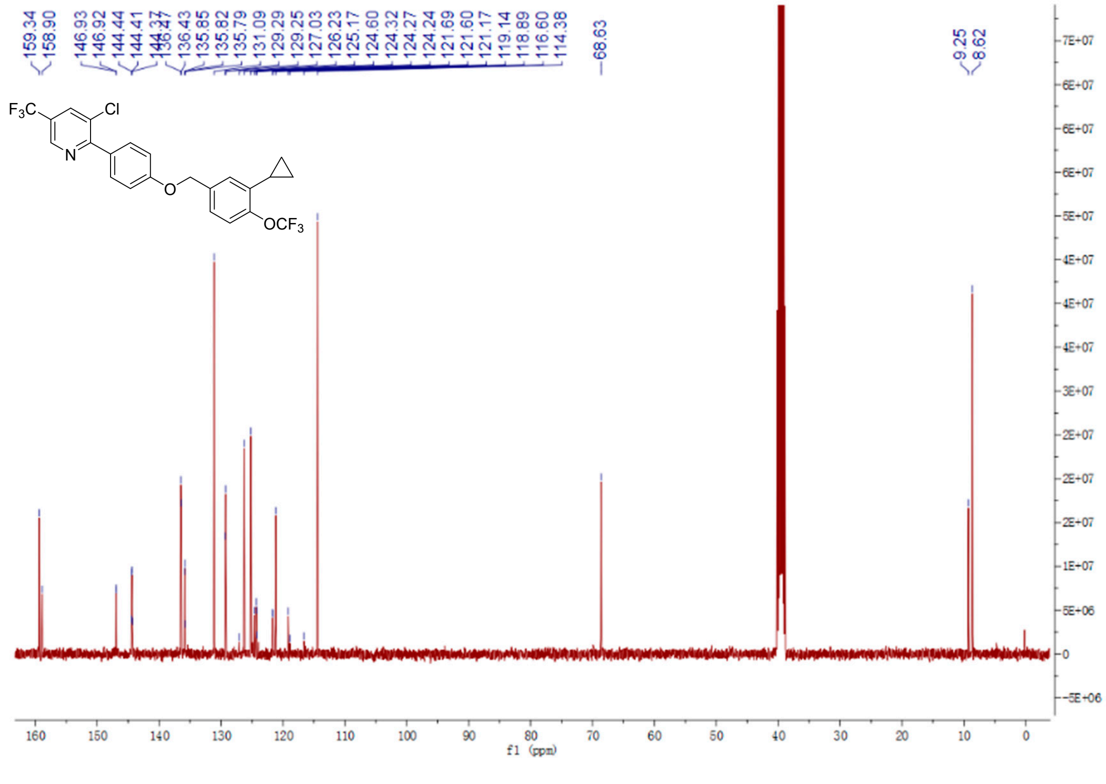

Figure S74. The ^{13}C NMR spectrum of **7q** (DMSO-d_6)

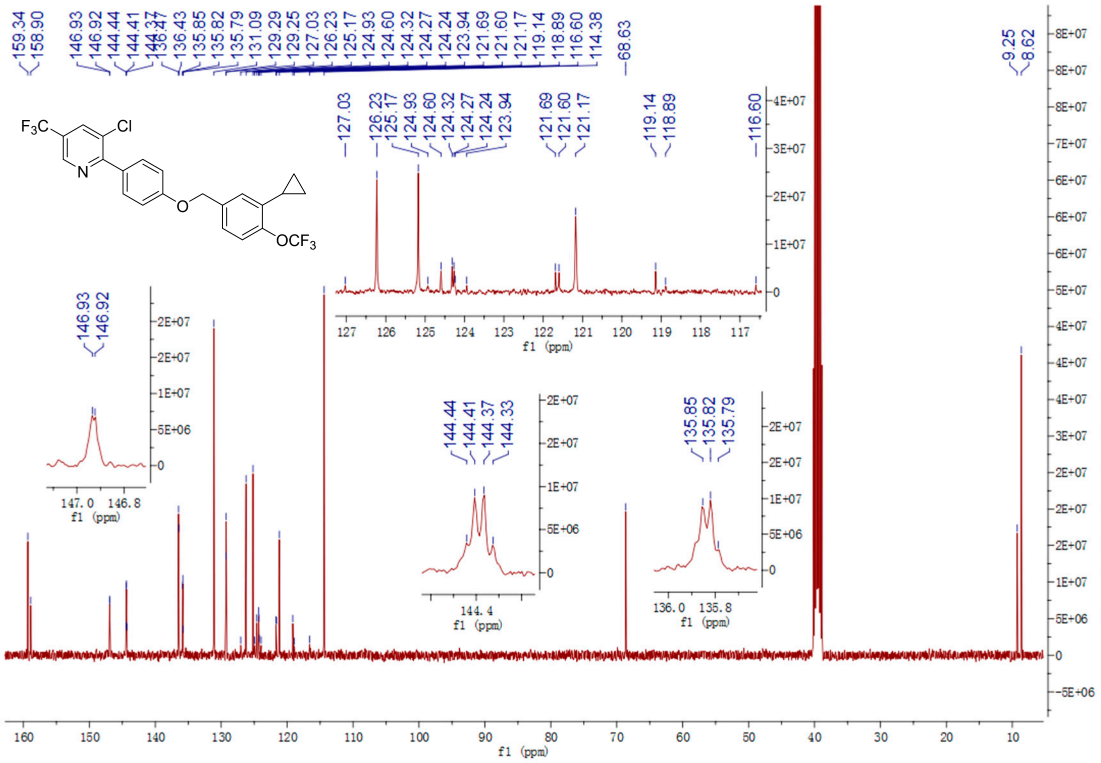

Figure S75. The ${}^{13}C$ NMR spectrum of **7q** (DMSO-d_6)

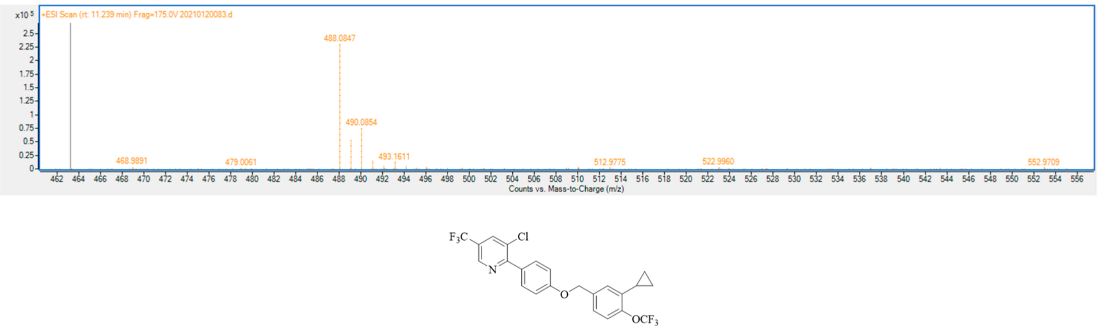

Figure S76. The HRMS spectrum of **7q**

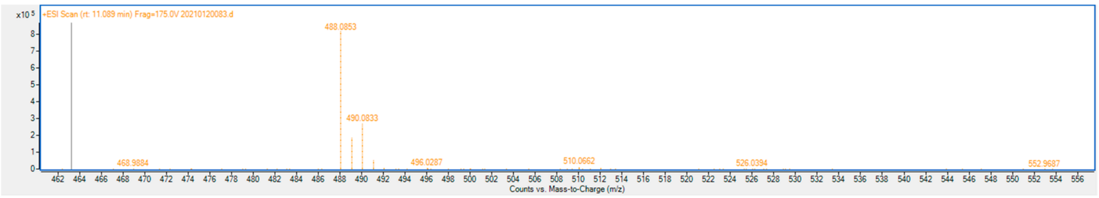

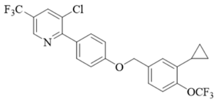

Figure S77. The HRMS spectrum of **7q**

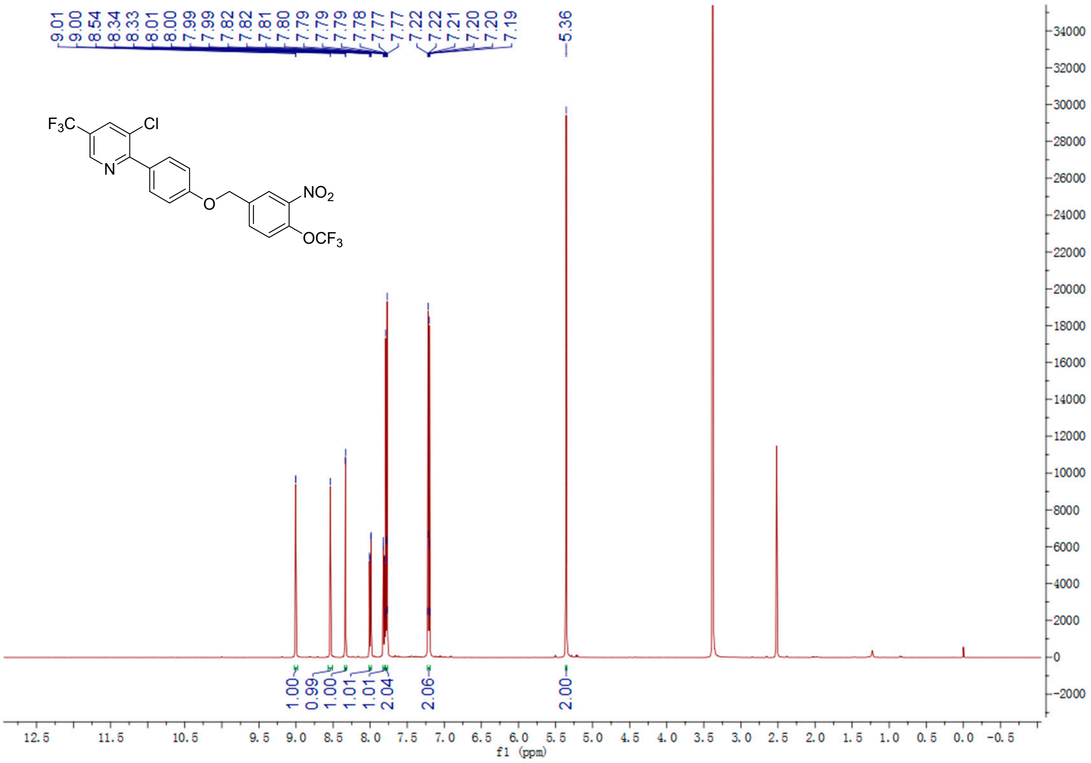

Figure S78. The ^{1}H NMR spectrum of **7r** (DMSO-*d*6)

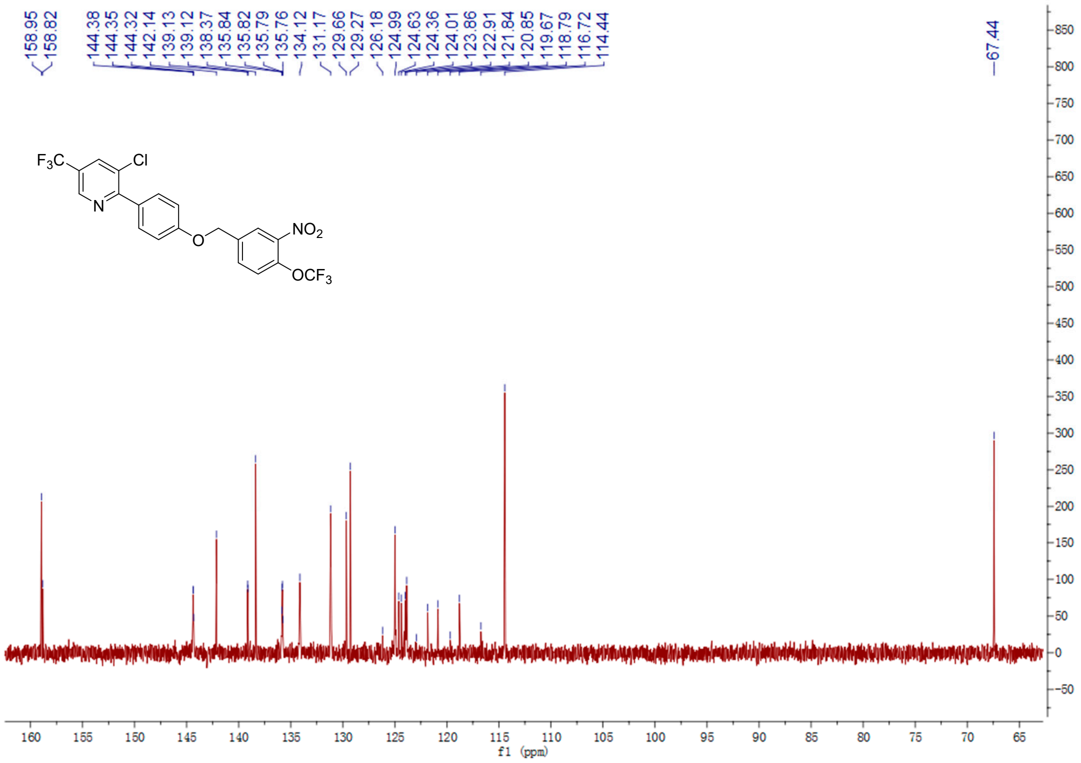

Figure S79. The ^{13}C NMR spectrum of **7r** (DMSO-d_6)

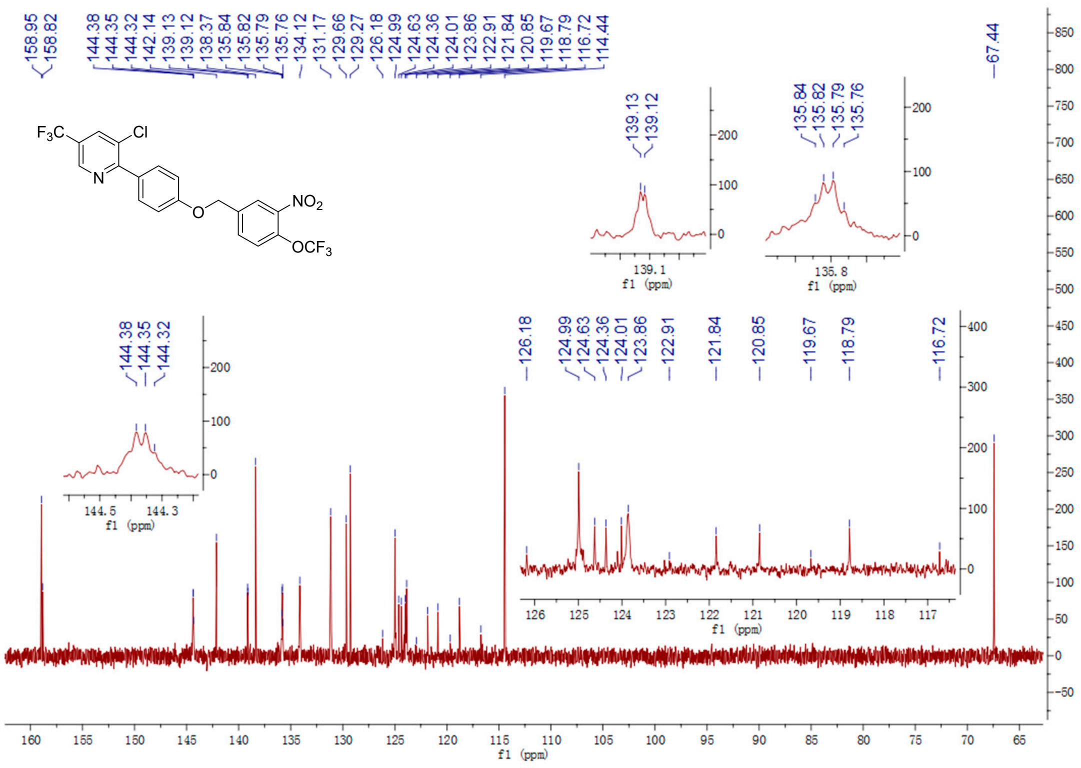

Figure S80. The ^{13}C NMR spectrum of **7r** (DMSO-d_6)

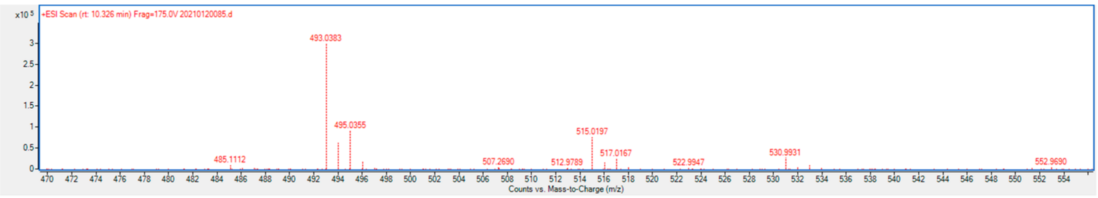

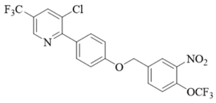

Figure S81. The HRMS spectrum of **7r**
